# Refined high-content imaging-based phenotypic drug screening in zebrafish xenografts

**DOI:** 10.1038/s41698-023-00386-9

**Published:** 2023-05-18

**Authors:** C. Sturtzel, S. Grissenberger, P. Bozatzi, E. Scheuringer, A. Wenninger-Weinzierl, Z. Zajec, J. Dernovšek, S. Pascoal, V. Gehl, A. Kutsch, A. Granig, F. Rifatbegovic, M. Carre, A. Lang, I. Valtingojer, J. Moll, D. Lötsch, F. Erhart, G. Widhalm, D. Surdez, O. Delattre, N. André, J. Stampfl, T. Tomašič, S. Taschner-Mandl, M. Distel

**Affiliations:** 1 St. Anna Children’s Cancer Research Institute (CCRI), Vienna, Austria; 2 Zebrafish Platform Austria for Preclinical Drug Screening (ZANDR), Vienna, Austria; 3 Faculty of Pharmacy, University of Ljubljana, Ljubljana, Slovenia; 4 Christian Doppler Laboratory for Advanced Polymers for Biomaterials and 3D Printing, TU Wien, Vienna, Austria; 5 Service d’Hématologie & Oncologie Pédiatrique, Timone Hospital, AP-HM, Marseille, France; 6 Centre de Recherche en Cancérologie de Marseille (CRCM), Aix-Marseille Université, CNRS, Inserm, Institut Paoli Calmettes, Marseille, France; 7 Department of Neurosurgery, Medical University of Vienna, Vienna, Austria; 8 Central Nervous System Tumors Unit, Comprehensive Cancer Center, Medical University of Vienna, Vienna, Austria; 9 Department of Molecular Oncology, Sanofi Research Center, Vitry-sur-Seine, France; 10 Renon Biotech and Pharma Consulting, Unterinn am Ritten (Bz), Italy; 11 Balgrist University Hospital, Faculty of Medicine, University of Zurich (UZH), Zurich, Switzerland; 12 INSERM U830, Diversity and Plasticity of Childhood Tumors Lab, PSL Research University, SIREDO Oncology Center, Institut Curie Research Center, Paris, France

**Keywords:** Cancer models, Phenotypic screening

## Abstract

Zebrafish xenotransplantation models are increasingly applied for phenotypic drug screening to identify small compounds for precision oncology. Larval zebrafish xenografts offer the opportunity to perform drug screens at high-throughput in a complex in vivo environment. However, the full potential of the larval zebrafish xenograft model has not yet been realized and several steps of the drug screening workflow still await automation to increase throughput. Here, we present a robust workflow for drug screening in zebrafish xenografts using high-content imaging. We established embedding methods for high-content imaging of xenografts in 96-well format over consecutive days. In addition, we provide strategies for automated imaging and analysis of zebrafish xenografts including automated tumor cell detection and tumor size analysis over time. We also compared commonly used injection sites and cell labeling dyes and show specific site requirements for tumor cells from different entities. We demonstrate that our setup allows us to investigate proliferation and response to small compounds in several zebrafish xenografts ranging from pediatric sarcomas and neuroblastoma to glioblastoma and leukemia. This fast and cost-efficient assay enables the quantification of anti-tumor efficacy of small compounds in large cohorts of a vertebrate model system in vivo. Our assay may aid in prioritizing compounds or compound combinations for further preclinical and clinical investigations.

## Introduction

Cancer is the second leading cause of death worldwide and remains a major global health challenge despite improvements in treatment^1^. Various strategies are pursued to develop novel small compounds with anti-cancer activity. Most often a target-based approach is applied, where small compounds are designed to inhibit the function of a protein based on protein structure. Predicted anti-tumor activity of such compounds is typically verified in cell culture assays, which are amenable to high-throughput drug screening. Subsequently, candidate molecules are tested in preclinical animal models. Here, mouse xenograft models, in particular patient-derived xenografts are the current gold standard to evaluate compound efficacy on whole organism level^2^. However, mouse xenografts are laborious, time-consuming and costly to establish, preventing their broad application in high-throughput drug screening.

In recent years, the zebrafish (*Danio rerio*) tumor cell xenotransplantation model has emerged as a complementary system to mouse xenografts^3^. Several groups demonstrated that larval zebrafish xenografts can be established with a variety of cancer cell lines ranging from leukemia to solid tumors^4–11^. More recently, even patient-derived xenografts for colorectal cancer and non-small cell lung carcinoma were generated in zebrafish and early data suggests that such zebrafish avatar models might be useful in predicting disease progression and in identifying patient-tailored treatments^12,13^.

Major advantages of the larval zebrafish xenograft model are the possibility to carry out a) live imaging e.g., of tumor cell proliferation and migration in the intact transparent vertebrate organism, b) fast and cost-efficient drug screening and c) toxicity evaluation of compounds on whole organism level at early developmental stages^14,15^. Larval zebrafish xenografts are small enough to fit into 96-well plates and compounds can be administered directly into the surrounding water. Such assays have been methodically improved and been increasingly used over the past years^4,11,16–20^. Furthermore, this format offers the opportunity to perform phenotypic drug screening on zebrafish xenografts at high-throughput. With phenotypic drug screening being superior in discovery of first-in-class compounds compared to target-based approaches zebrafish-based screens promise to yield novel treatment strategies^21^.

Different imaging setups (wide field, confocal) have been employed towards automated imaging of zebrafish xenografts including a pioneering work using a high-content imager, but so far these have not been broadly applied likely due to remaining technical challenges^11,22,23^.

Here, we provide a refined and robust workflow for high-content imaging of larval zebrafish xenografts. We reinvestigated the suitability of different injection sites and dyes for various cell lines from different tumor entities. In addition, we describe embedding methods for easy high-content imaging. Furthermore, we show options for automated recognition of zebrafish larvae and tumor cells and automated quantification of tumor size over consecutive days by adapting commercially available high-content imaging software.

Our setup allows us to test the in vivo efficacy of compounds within only one week of time. Furthermore, we show that not only cell lines of various tumor entities, but also patient-derived cells can be transplanted and grow in zebrafish larvae, providing the opportunity to evaluate personalized therapy responses in a clinically relevant time span.

Our workflow for high-content imaging-based drug screening and the established comprehensive compendium of zebrafish xenograft cancer models will serve as a valuable resource for preclinical compound testing and can easily be adapted for additional tumor entities.

## Results

### Comparison of commonly used injection sites

**Fig. 1:**
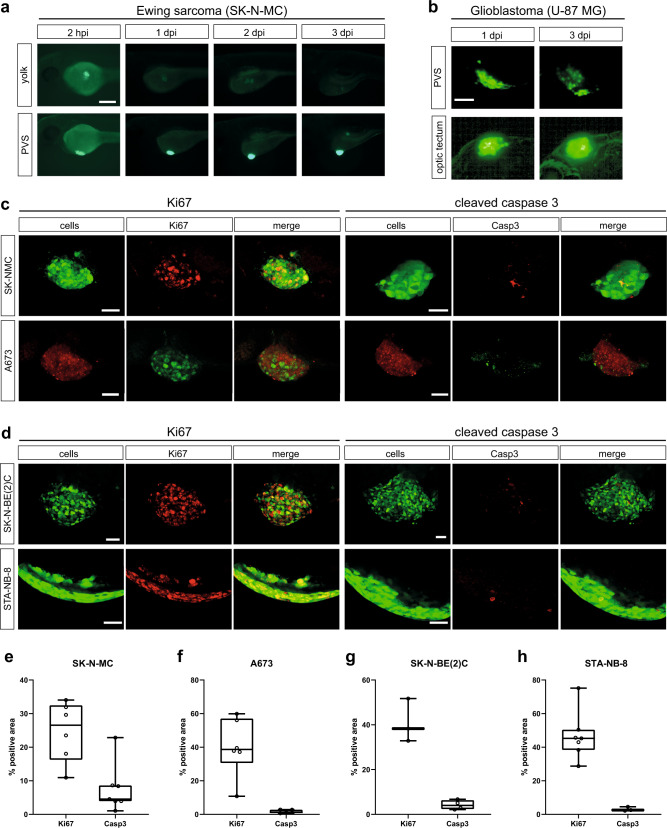
Characterization of pediatric solid tumor xenografts. **a** GFP-labeled SK-N-MC Ewing sarcoma cells (shSK-E17T) were transplanted into 2 dpf old zebrafish embryos. Injection was performed into the yolk (*n* = 9) vs. the PVS (*n* = 5) and embryos were monitored with a fluorescence miscroscope (Axio Zoom.V16, Zeiss) daily until 3 dpi. Scale bar is 250 µm. **b** GFP-labeled U-87 MG glioblastoma cells were transplanted into the PVS vs. the optic tectum (orthotopic) and imaged at 1 dpi and 3 dpi using the Operetta CLS. Scale bar is 100 µm. **c** Embryos transplanted with SK-N-MC or A673 Ewing sarcoma cells were fixed at 3 dpi and immunostained for Ki67 (SK-N-MC *n* = 6, A673 *n* = 6) and cleaved caspase 3 (SK-N-MC *n* = 7, A673 *n* = 5). Scale bars are 50 µm. **d** Embryos transplanted with SK-N-BE(2)C and STA-NB-8 neuroblastoma cells were fixed at 3 dpi and immunostained for Ki67 (SK-N-BE(2)C *n* = 3, STA-NB-8 *n* = 7) and cleaved caspase 3 (SK-N-BE(2)C *n* = 4, STA-NB-8 *n* = 3). Immunostained larvae were imaged with a confocal microscope (Leica SP8). Scale bars are 50 µm. **e**–**h** Quantification of percentages of Ki67-/cleaved Caspase 3-positive areas of immunostained xenografted tumors (SK-N-MC, A673, SK-N-BE(2)C, and STA-NB-8) was performed with ImageJ and plotted with Tukeys box plots. Line represents the median value, box spans 25th to 75th percentile, and whiskers span 5th to 95th percentile.

To evaluate location specific effects towards persistence of tumor cells in zebrafish and the ability to image the cells, we compared two commonly used injection sites, yolk and perivitelline space (PVS). With a main focus on pediatric tumors and cell lines we initially investigated, if SK-N-MC Ewing sarcoma cells can survive and proliferate in the zebrafish embryo upon xenotransplantation at 2 days post fertilization (dpf). To test this, we used the GFP-expressing SK-N-MC derivative cell line shSK-E17T^24^. We injected approximately 200–400 cells into the yolk or PVS, and monitored xenografted embryos for 3 consecutive days. Whereas cell numbers decreased in the yolk already after 24 h post injection (hpi) and were hardly recognizable after 3 days post injection, SK-N-MC cells transplanted into the PVS persisted and increased in numbers (Fig. 1a). At 2 hpi we found tumor cells to be dispersed at the PVS injection site, but to form a compact mass by 1 day post injection (dpi) (Supplementary Figure 1a), which increased in size over subsequent days (Fig. 1a). Ki67 immunofluorescence staining confirmed that GFP-expressing SK-N-MC as well as RFP-expressing A673 Ewing sarcoma cells proliferate in zebrafish embryos/larvae up to 7 dpi, the latest time point we monitored (Fig. 1c, left panel and Supplementary Figure 1b). Staining for activated Caspase 3 showed hardly any apoptotic cells (Fig. 1c, right panel). Furthermore, injection of Ewing sarcoma cells into transgenic larvae with labeled vasculature *Tg(kdrl:Hsa.HRAS-mCherry)*^*s896*^ revealed angiogenesis towards tumor masses at 3 dpi (Supplementary Figure 1c). These results indicated that the PVS is a suitable injection site for Ewing sarcoma cells and likely other solid tumor cells. In addition to Ewing sarcoma cells, we also tested PVS engraftment of a neuroblastoma cell line with *MYCN* oncogene amplification, SK-N-BE(2)C (engineered to express H2B-GFP) and a patient-derived culture of neuroblastoma bone marrow metastasis, STA-NB-8 (*MYCN* amplified, *ALK* mutated). Similar to SK-N-MC and A673, SK-N-BE(2)C formed a compact tumor cell cluster, however, STA-NB-8 stayed dispersed and crescent-shaped at the injection site. SK-N-BE(2)C and STA-NB-8 were positive for Ki67 in zebrafish and showed almost no activated Caspase 3, indicating that the PVS is a permissive injection site (Fig. 1d). Quantification of immunofluorescence staining confirmed a comparable level of Ki67-positive cells across tested tumor types, as well as low numbers of cells positive for cleaved Caspase 3 (Fig. 1e-h). However, for selected brain tumor cell lines, we noted that injection into the PVS did not support tumor cell maintenance. We used GFP-expressing U-87 MG glioblastoma cells to compare PVS to orthotopic injection into the brain (optic tectum). While cells decreased in the PVS, we observed that the optic tectum environment maintained U-87 MG cells and we quantified a slight increase (1.2-fold) in tumor size from 1 dpi to 3 dpi (Fig. 1b, Supplementary Figure 2).

### Automated high-content imaging of zebrafish xenografts

**Fig. 2:**
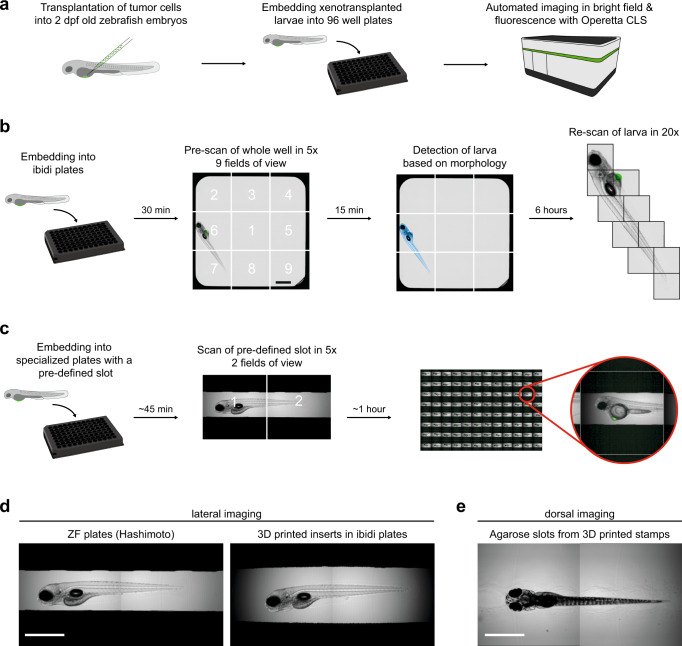
Embedding & automated imaging of xenotransplanted zebrafish larvae. **a** Workflow: Zebrafish larvae are xenotransplanted with tumor cells and embedded into 96-well plates and automatically imaged with the Operetta CLS high-content imaging system. **b** Embedding of xenotransplanted larvae into ibidi view plates and imaging with prescan & rescan function of the Operetta CLS: First the whole well is imaged in 9 fields of view with a 5x air objective, then the xenotransplanted larva is detected (blue). Finally the area of interest is rescanned at higher magnification with a 20x water objective. **c** Embedding of xenotransplanted larvae into 96-well plates with a pre-defined slot for zebrafish. Imaging of 2 fields of view with a 5x air objective. **d** For lateral imaging larvae can be embedded into ZF plates (left) or ibidi plates with 3D-printed inserts (right). Scale bar is 1 mm. **e** For dorsal imaging agarose stamps (adapted from Wittbrodt et al.^25^) were produced that create slots for zebrafish larvae. Scale bar is 1 mm.

In order to screen small compounds for tumor-growth-inhibiting effects at medium to high-throughput, we next sought to automate image acquisition and image analysis of xenografted zebrafish larvae enabling reproducible quantification of tumor sizes at different time points. Adapting a cell-based drug screening setup, we applied a high-content imager (Operetta CLS, PerkinElmer) for automated image acquisition of xenografted zebrafish larvae in 96-well plate format (Fig. 2a).

Proper positioning of zebrafish larvae is crucial for time-efficient imaging. Initially, we imaged zebrafish xenografts in commercial square bottom plates (ibidi), well suited for high-content imaging of cells in 2D. To image only the areas where zebrafish larvae are located inside the well, we applied a prescan/rescan strategy. We initially recorded the wells at low magnification (5x objective) to obtain an overview image. We trained the Operetta CLS Harmony software to detect the zebrafish in the well based on differences in texture in the image of the brightfield channel, and only re-imaged the areas covered by the larva at higher magnification for detailed images (20x) (Fig. 2b). This strategy enabled automated image acquisition at high resolution of considerable sample numbers in a reasonable time frame.

We also explored options for further reducing the imaging area within the wells even at lower magnification. Here, we aimed for reproducibly positioning zebrafish in the wells by using imaging plates with an insert, which keeps zebrafish centric in the well. To achieve this, we used 96-well imaging plates with pre-defined slots for zebrafish larvae (ZF plates, Hashimoto) (Fig. 2c). This setup allowed us to record images of xenografted larvae in brightfield and fluorescence in confocal quality at higher throughput. Typical imaging time for one plate with 96 larvae was around 60 min (5x air objective, 2 fields of view, 23 planes per larva, brightfield and one fluorescence channel). As the original ZF plates only allowed for imaging with 5x air objectives due to their thick glass bottom, we also explored alternatives and developed 3D-printed inserts customized for commercially available high-content imaging plates like ibidi view plates (Fig. 2d, Supplementary Figure 3a). As some zebrafish xenografts like orthotopic brain injections might require dorsal imaging, we also adapted an existing protocol to 3D print stamps that produce slots in agarose for dorsal positioning of larvae in imaging plates (Fig. 2e, Supplementary Figure 3b)^25^.

In summary, we provide robust setups that allow for fast and reproducible lateral or dorsal automated high-content imaging of (xenografted) zebrafish larvae.

### Automated tumor detection and tumor size quantification

**Fig. 3:**
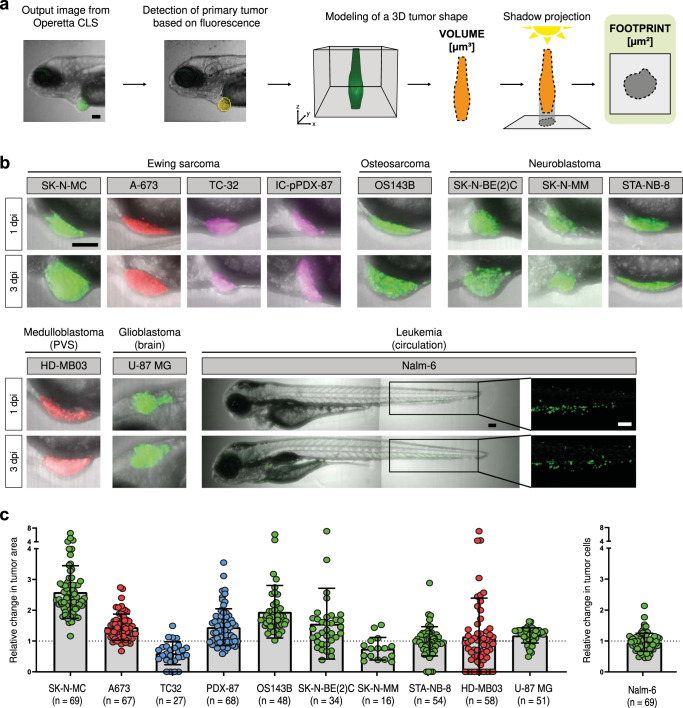
Automated quantification of tumor size. **a** Workflow: Initially, the Harmony software detects the large tumor cell mass based on fluorescence. Then images from the z-stacks are modeled into a 3D tumor shape ( = tumor volume in µm³). This 3D shape is then projected back onto a 2D plane creating a footprint of the tumor (tumor footprint in µm²). **b** Representative images for transplanted tumor entities (SK-N-MC (SKshctrl), A-673, TC-32, IC-pPDX-87, OS143B, SK-N-BE(2)C, SK-N-MM, STA-NB-8, HD-MB03, U-87 MG, Nalm-6) at 1 dpi and 3 dpi. **c** Dot plots show relative change in tumor area (3 dpi/1 dpi) for transplanted tumor entities (SK-N-MC, A-673, TC32, IC-pPDX-87, OS143B, SK-N-BE(2)C, SK-N-MM, STA-NB-8, HD-MB03, U-87 MG) and relative change in tumor cells (Nalm-6). Error bars represent SD. Scale bars are 100 µm.

To automate tumor cell detection and quantification of the size of the tumor cell cluster, we adopted analysis modules of the Harmony software (PerkinElmer) for our specific application in zebrafish (Fig. 3a). First, tumor detection was performed according to fluorescence intensity. Common thresholds were set according to the signal strength of individual cell lines to ensure proper tumor detection. We compared two different analysis methods to evaluate tumor size, both based on recording a 3D stack of the tumor: volume and footprint area analysis. While actual volume measurements are theoretically superior to area measurements of objects with complex shapes, we encountered practical limitations for precise volume measurements. Even in confocal mode we observed significant scattering around the non-transparent tumor cell mass resulting in a blurred halo, especially along the z-direction. Indeed, when we compared calculated volumes of almost spherical tumors with our measurements, we found that measured volumes were significantly overestimating actual volumes (5- to 6-fold) (Supplementary Figure 4). Alternatively, we projected the tumor shape into a single plane (footprint projection) and measured tumor area. This footprint projection reliably provided measurements in good agreement with our observations for spherical tumors.

Hence, we subsequently applied footprint projection to measure the tumor size in xenografted zebrafish (Fig. 3c).

### Automated quantification can be applied across tumor entities

Next, we investigated if different cancer cell types with diverse genetic background (Table 1) would persist and would be quantifiable when xenografted into zebrafish larvae with our analysis. Using our established setup, we xenografted various cell lines from different, mostly pediatric, tumor entities including Ewing sarcoma, neuroblastoma, osteosarcoma, medulloblastoma, glioblastoma and acute lymphoblastic leukemia. For Ewing sarcoma we also generated a xenograft model from patient-derived cells, which had been passaged through a mouse PDX model (IC-pPDX-87^26^,) and for neuroblastoma from a short-term 2D culture (STA-NB-8) (Fig. 3b, c). All xenotransplanted larvae were imaged at 1 dpi and 3 dpi. Subsequently, the relative change in tumor size (3 dpi/1 dpi) was calculated to determine changes over the 48 h time span (>1: increase; =1: stagnation; <1: decrease). Among Ewing sarcoma cell lines, SK-N-MC (SKshctrl) cells increased 2.6-fold on average whereas A-673 cells had a slower growth rate with an increase in area of about 1.4-fold. TC32 Ewing sarcoma cells decreased in tumor area (relative change: 0.6-fold), while patient-derived-cells IC-pPDX-87 expanded 1.4-fold. Transplanted OS143B osteosarcoma cells increased 2-fold in size. Within the neuroblastoma panel *MYCN* amplified SK-N-BE(2)C cells engrafted best with a 1.5-fold increase over 48 h. SK-N-MM cells, which carry *ATRX* whole exon deletions resulting in in-frame fusions (*ATRX*^mut^), decreased slightly over time (relative average change: 0.75-fold) and the PDX STA-NB-8 (*MYCN* amplified, *ALK* mutated) maintained their size (1-fold change). To exclude that the differences in engraftment and growth among the three tested neuroblastoma cultures is caused by maintaining zebrafish xenografts at 34 °C, we investigated in vitro growth rates at 34 °C and 37 °C, but did not observe any significant difference (Supplementary Figure 5). In xenografts with the medulloblastoma cell line HD-MB03 a small increase in tumor area with a relative change of 1.1-fold could be assessed over the course of 48 h. U-87 MG glioblastoma cells injected orthotopically into the brain showed a relative increase in tumor area of 1.2-fold.

**Table 1:** Cell lines and patient-derived cells used in this study.

Cell line	Tumor type	Treatment^a^	Clinical time point	Age at diagnosis	Sex	Genetic subtype
SK-N-MC	Ewing sarcoma	Radiotherapy, chemotherapy (VC; A)	Metastasis at relapse	12 years^b^	F	Type I, TP53^mut^, STAG2^mut^
A-673	Ewing sarcoma	–	Primary at diagnosis	15 years	F	Type I, CDKN2A^del^, TP53^mut^, BRAF^mut^
TC32	Ewing sarcoma	–	Primary at diagnosis	31 months	F	Type I, CDKN2A^del^, STAG2^mut^
IC-pPDX-87	Ewing sarcoma	1st line: VDCIE2nd line: TI	Primary at relapse	13 years^b^	M	Type I, CDKN2A^del^, TP53^mut^
OS143B	Osteosarcoma	–	Primary at diagnosis	13 years	F	HOS + KRAS^mut^
SK-N-BE(2)C	Neuroblastoma (INSS 4/high-risk)	–	Metastasis (bm) at diagnosis	26 months	M	MYCN^amp^
SK-N-MM	Neuroblastoma (INSS 4/high-risk)	n.a.	Metastasis (bm)	8 years	F	ATRX^del^
STA-NB-8^c^	Neuroblastoma (INSS 4/high-risk)	–	Metastasis (bm) at diagnosis	28 months	F	MYCN^amp^, ALK^F1174L^
U-87 MG	Glioblastoma	n.a.	n.a.	n.a.	M	IDH^wt^, NF1^mut^, PTEN^mut^
HD-MB03	Medulloblastoma		Primary at diagnosis	3 years	F	Group 3
Nalm-6	ALL	n.a.	Relapse	19 years^b^	M	–

^a^*V* Vincristin, *C* Cyclophosphamide, *A* Actinomycin D, *D* doxorubicin, *I* Ifosfamide, *E* Etoposide, *T* Temozolomide, *I* Irinotecan, *bm* bone marrow.

^b^Age at sampling.

^c^Low passage patient-derived cell line.

In addition to solid tumor detection, we also automated the detection of acute lymphoblastic leukemia cells injected into circulation of zebrafish larvae. Previously, we have demonstrated that Nalm-6 cells in zebrafish larvae stain positive for Ki67 and we analyzed cell numbers by manual counting^27^. Here, applying a spot count algorithm of the Harmony software we were able to determine cell numbers in an automated fashion. The analysis shows that leukemic Nalm-6 cells are maintained over 48 h in zebrafish larvae without any changes (relative change: 1-fold).

### CellTrace Violet is superior to CM-DiI for labeling tumor cells in zebrafish xenografts

**Fig. 4:**
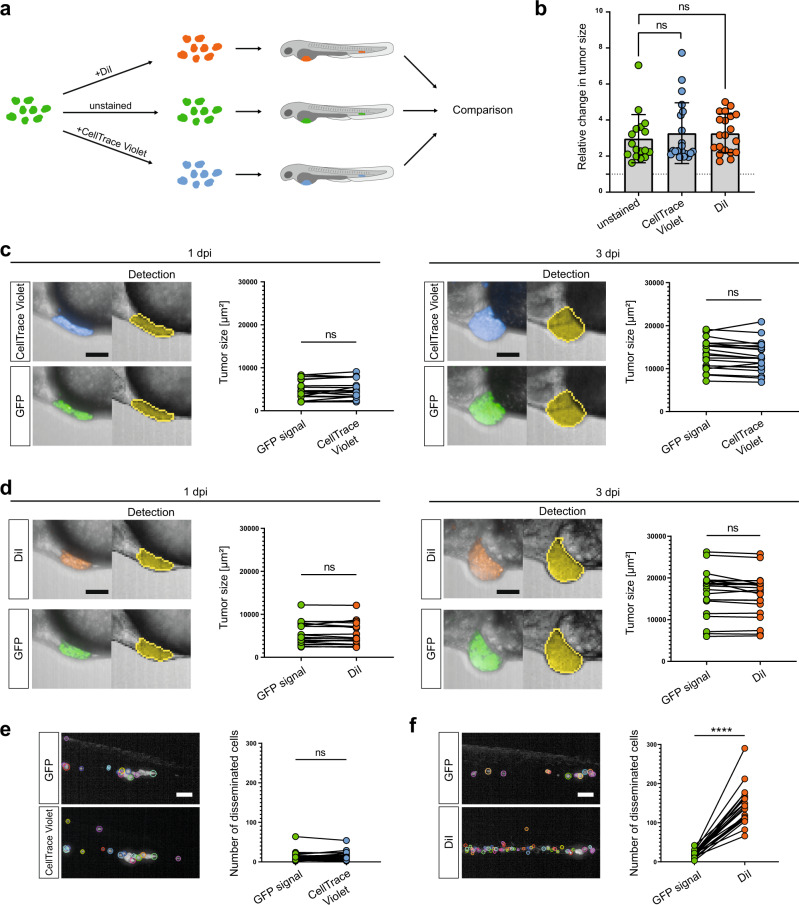
Comparison of CellTracker^TM^ CM-DiI and CellTrace^TM^ Violet as cell dyes for xenotransplantation. **a** Overview: GFP-labeled SK-N-MC cells (SKshctrl) were counter-labeled either with CellTracker^TM^ CM-DiI or CellTrace^TM^ Violet. **b** Dot plot shows relative change in tumor size in larvae transplanted with unstained cells (*n* = 17) or cells labeled with DiI (*n* = 20) or CellTrace^TM^ Violet (*n* = 20). **c** Comparison of tumor detection based on either GFP- or CellTrace^TM^ Violet-signal. Dot plots show detected tumor areas at 1 dpi and 3 dpi. **d** Comparison of tumor detection based on either GFP- or DiI-signal. Dot plots show detected tumor areas at 1 dpi and 3 dpi. **e**, **f** The number of disseminated tumor cells at 3 dpi was determined with a spot count algorithm. Statistical analysis was performed with a Kruskal–Wallis test in (**b**) and Mann–Whitney tests in (**c**–**e**) (ns: not significant, ****p < 0.0001). Error bars represent SD. Scale bars are 100 µm.

Not all tumor cells, especially low passage primary or patient-derived cells carry an intrinsic fluorescent label/express fluorescent proteins. Thus, dyes to reliably label such cells for automated imaging are needed. We compared CellTrace^TM^ Violet with CellTracker^TM^ CM-DiI (DiI), the most commonly used dye in zebrafish xenografts to see whether they are suitable for our automated setup. For validation we labeled GFP-expressing SK-N-MC (SKshctrl) Ewing sarcoma cells with either CellTracker^TM^ CM-DiI or CellTrace^TM^ Violet (Fig. 4a). CellTracker^TM^ CM-DiI is a lipophilic membrane dye, while CellTrace^TM^ Violet is a cytoplasmic stain. It belongs to the class of succinimidyl esters and binds free amines within the cells^28^. No significant difference in tumor growth in unlabeled cells compared to cells either labeled with DiI or CellTrace^TM^ Violet could be detected when tumors were analyzed based on their GFP fluorescence, indicating that none of the dyes affects proliferation at applied concentrations (Fig. 4b). Also, no significant difference in detection of primary tumors at 1 dpi or 3 dpi with both dyes was recognized (Fig. 4c, d).

Next, we sought to compare the quantification of cells that have migrated out of the primary tumor site after injection. Here, we applied two different analysis methods: 1) a spot count algorithm and 2) quantification of fluorescent area outside the primary tumor. With the spot count analysis CellTrace^TM^ Violet was reliably staining GFP-positive tumor cells in circulation. In contrast, with DiI significantly more objects were detected compared to the GFP signal (Fig. 4e, f, Supplementary Figure 6a, b). In general, we often detected DiI-labeled particles in the caudal hematopoietic tissue even in the complete absence of GFP-positive cells in this region (Supplementary Figure 6c). The same trend was observed by quantification of the fluorescent area. When cells were labeled with CellTrace^TM^ Violet only a slightly larger area of disseminated cells was detected (Supplementary Figure 6d). This small discrepancy was mainly caused by the autofluorescence of the otolithes (Supplementary Figure 6e). However, when cells were labeled with DiI a substantially higher amount of fluorescent area was recognized outside the primary tumor region in the tail (Supplementary Figure 6f). The deviation from the GFP signal was significantly higher in DiI- vs. CellTrace^TM^ Violet-labeled cells, suggesting erroneous results with DiI labeling (Supplementary Figure 6g).

### Fast testing of targeted compounds at high-throughput in vivo

To demonstrate that our setup is well suited for single and combination drug testing we performed a proof-of-principle small compound screen using molecules targeted against Ewing sarcoma, osteosarcoma, glioblastoma, and neuroblastoma. For treatment of Ewing sarcoma xenografts we chose YK-4–279, described as inhibitor of EWS::FLI1/RNA helicase A interactions and microtubule polymerization, which has been reported to induce apoptosis in Ewing sarcoma cells and lead to growth reduction in a mouse xenograft model^29,30^. For Ewing sarcoma as well as osteosarcoma it has recently been reported that the YAP/TEAD axis could serve as a potential target^31,32^. Therefore, we investigated the TEAD inhibitor K-975 for Ewing sarcoma and osteosarcoma treatment in our study^33^.

Recently, inhibitors of anti-apoptotic proteins gained attention as alternative therapy target, hence we tested the BCL-X_L_ inhibitor A-1331852 against orthotopically injected glioblastoma xenografts.

**Fig. 5:**
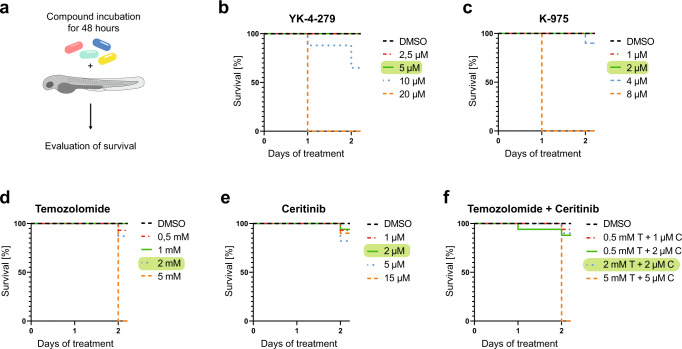
No observed effect concentration (NOEC) determination. NOEC was determined as shown in **a** for YK-4-279 (**b**), K-975 (**c**), Temozolomide (**d**), Ceritinib (**e**), and for the combination of Temozolomide + Ceritinib (**f**). Larvae were incubated with different concentrations of compounds or DMSO (YK-4-279: DMSO (*n* = 29), 2,5 µM (*n* = 34), 5 µM (*n* = 41), 10 µM (*n* = 34), and 20 µM (*n* = 28); K-975: DMSO (*n* = 11), 1 µM (*n* = 13), 2 µM (*n* = 11), 4 µM (*n* = 11) and 8 µM (*n* = 10); Temozolomide: DMSO (*n* = 9), 0,5 mM (*n* = 16), 1 mM (*n* = 14), 2 mM (*n* = 16) and 5 mM (*n* = 15); Ceritinib: DMSO (*n* = 9), 1 µM (*n* = 15), 2 µM (*n* = 16), 5 µM (*n* = 17) and 15 µM (*n* = 10); Ceritinib + Temozolomide: DMSO (*n* = 9), 1 µM C + 0.5 mM T (*n* = 17), 2 µM C + 0.5 mM T (*n* = 17), 2 µM C + 2 mM T (*n* = 10) and 5 µM C + 5 mM T (*n* = 20)) and survival was evaluated after 48 h. Determined NOECs were 5 µM for YK-4-279, 2 µM for K-975, 2 mM for Temozolomide, 2 µM for Ceritinib, and 2 mM Temozolomide + 2 µM Ceritinib for the combination (highlighted in green).

Xenografts with neuroblastoma cells were treated with the chemotherapeutic drug Temozolomide (under clinical investigation for treatment of refractory/relapsed neuroblastoma: NCT02308527 or NCT01467986) and the ALK inhibitor (ALKi) Ceritinib^34,35^. Aiming to treat zebrafish xenografts at maximum tolerated concentrations, the “No Observed Effect Concentrations” (NOECs) of YK-4-279, K-975, Temozolomide, Ceritinib and the combination of Temozolomide plus Ceritinib were determined. Here, we scored overall survival and developmental abnormalities including edema formation (Fig. 5a). NOECs were assessed under identical experimental conditions (age of larvae, start and duration of treatment, temperature) as in the xenograft setting and were found to be 5 µM for YK-4-279, 2 µM for K-975, 2 mM for Temozolomide, 2 µM for Ceritinib and 2 mM Temozolomide + 2 µM Ceritinib for their combination (Fig. 5b–f). The NOEC for A-1331852 (10 µM) was already determined in a previous study, where we identified dual MCL-1/BCL-X_L_ inhibition to be highly efficient against EwS xenografts^36^.

**Fig. 6:**
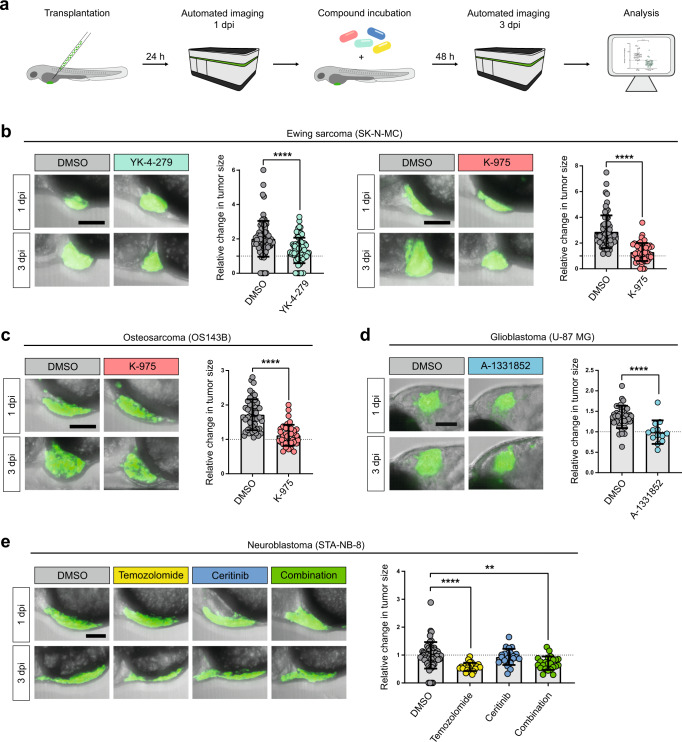
Treatment of xenografted zebrafish larvae with targeted compounds. **a**) Workflow: Larvae are xenotransplanted at 2 dpf. After 24 h (1 dpi) they are anesthetized, embedded and automatically imaged in the Operetta CLS. Larvae are then incubated with compounds at NOEC concentration or DMSO as a control for 48 h. Larvae are imaged again on the Operetta CLS (3 dpi) and tumor sizes are quantified and analyzed. **b**) Treatment of larvae xenotransplanted with GFP-labeled SK-N-MC cells (shSK-E17T) with 5 µM YK-4-279 (*n* = 73) or DMSO (*n* = 78) (left panel) and treatment of GFP-labeled SK-N-MC cells (SKshctrl) with 2 µM K-975 (*n* = 54) or DMSO (*n* = 70) (right panel). **c**) Treatment of larvae xenotransplanted with GFP-labeled OS143B cells with 2 µM K-975 (*n* = 39) or DMSO (*n* = 52). **d**) Treatment of larvae xenotransplanted with GFP-labeled U-87 MG cells with 10 µM A-1331852 (*n* = 13) or DMSO (*n* = 38). **e**) Treatment of larvae xenotransplanted with GFP-labeled STA-NB-8 patient-derived cells with 2 mM Temozolomide (*n* = 24), 2 µM Ceritinib (*n* = 22), 2 mM Temozolomide + 2 µM Ceritinib (*n* = 23) or DMSO (*n* = 54). Scale bars are 100 µm. Dot plots show relative change in tumor size (3 dpi/1 dpi). Statistical analyses were performed with a Mann–Whitney test in (**b**–**d**) and with a Kruskal–Wallis test in (**e**) (****p < 0.0001, **p < 0.005). Error bars represent SD.

For compound testing, xenotransplanted larvae were imaged at 1 dpi followed by 48 h of compound treatment. At 3 dpi larvae were imaged again and tumor sizes were evaluated (Fig. 6a). The growth of SK-N-MC Ewing sarcoma xenografts was significantly decreased when treated with YK-4-279 from 2-fold to 1.3-fold. This effect was shown to be dose-dependent (Supplementary Figure 7). In addition, K-975 also caused a tumor growth halt down to 1.1 fold (Fig. 6b). Similarly, in osteosarcoma xenografts application of 2 µM K-975 led to a decrease (1.7-fold to 1-fold) in tumor growth (Fig. 6c).

Glioblastoma xenografts incubated in A-1331852 containing medium were significantly smaller (1-fold change) than the DMSO-treated group (1.3-fold increase (Fig. 6d).

For Neuroblastoma cell line STA-NB-8 (*ALK*^F1174L^) we found in vitro that it is more sensitive to Temozolomide and Ceritinib than SK-N-MM or SK-N-BE(2)C (both *ALK*^wt^) (Supplementary Figure 8a, b). In zebrafish, we could observe significant reduction in tumor area of Temozolomide-treated STA-NB-8 xenografts, whereas Ceritinib had no significant effect in vivo. Combination treatment of Temozolomide and Ceritinib did not show any enhanced effects (Fig. 6d). In contrast, *ALK*^wt^ xenografts showed no significant response upon single agent treatment and either no (SK-N-BE(2)C) or a small response upon combination treatment (SK-N-MM) (Supplementary Figure 8c, d).

**Fig. 7:**
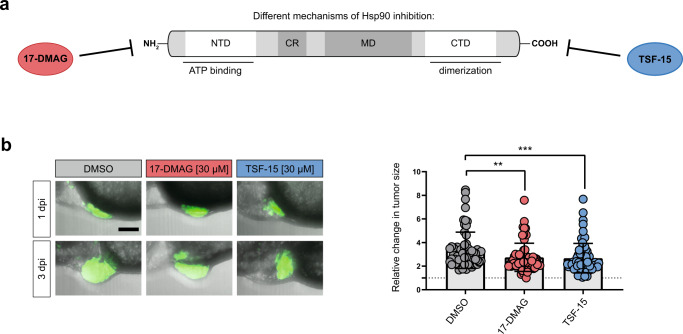
Treatment of SK-N-MC xenografted zebrafish larvae with Hsp90 inhibitors. **a** Schematic representation of different mechanisms of Hsp90 inhibition. **b** Treatment of larvae xenotransplanted with GFP-labeled SK-N-MC cells (SKshctrl) with 30 µM 17-DMAG (*n* = 65), 30 µM TSF-15 (*n* = 68) or DMSO (*n* = 65). Scale bar is 100 µm. Dot plots show relative change in tumor size (3 dpi/1 dpi). Statistical analysis was performed with a Kruskal–Wallis test (***p < 0.001, **p < 0.01). Error bars represent SD.

Zebrafish xenografts also promise to be a fast in vivo anti-tumor efficacy verification model for completely novel compounds. To showcase such an application, we investigated newly synthesized Hsp90 inhibitors. Hsp90 serves as a chaperone ensuring the proper folding of more than 400 client proteins, with many of them involved in oncogenesis. Interestingly, EWS::FLI1, the driver of EwS is a client itself and previous data reported efficacy of an Hsp90 inhibitor against EwS cells^37^. Most current Hsp90 inhibitors are targeted against the N-terminal domain (e.g., 17-DMAG, Fig. 7a) and suffer from induction of heat shock response (HSR), which limits their clinical efficacy. To overcome this problem, we designed allosteric C-terminal Hsp90 inhibitors, which should not cause HSR (e.g., TSF-15, Fig. 7a, Supplementary Figure 9). After in vitro tests against EwS cells (Supplementary Figure 11), we evaluated promising compounds in EwS zebrafish xenografts and observed an in vivo activity for TSF-15 similarly to 17-DMAG at comparable concentrations, indicating that this compound is a starting point for further structural optimization (Fig. 7b).

Ultimately, this illustrates that our setup is suitable for preclinical screening of newly synthesized compounds and previously reported targeted therapies as single agents as well as in combination with a chemotherapy backbone for pediatric tumor entities within the short time span of one week.

## Discussion

Larval zebrafish xenografts are gaining increasing attention in cancer research and precision oncology as a vertebrate model system complementary to mouse xenografts^18^. Zebrafish larvae are well suited for small compound screening and several approaches towards automating key steps in the drug screening workflow have been undertaken, including applying automated high-content imaging^19^. Despite successful proof-of-principles of high-content imaging-based analysis of zebrafish xenografts^11,19^, this approach has not been widely adopted subsequently and the potential of zebrafish xenografts for the discovery of new therapeutic compounds by automated high-throughput high-content screening has not been fully exploited yet. Here, we provide a robust protocol for high-content imaging-based analysis of zebrafish xenografts, which may find wide application for different tumor entities. We already demonstrated the reliability of our workflows for identifying new drug combinations with high efficacy against Ewing sarcoma^36^. Especially the combination of irinotecan with anti-apoptotic protein inhibitors targeted against MCL-1 and BCL-X_L_ or dual MCL-1/BCL-X_L_ was highly efficient^36^.

In addition, we established and characterized a comprehensive compendium of zebrafish xenograft cancer models with different pediatric tumor cell lines, which can be applied in preclinical compound testing.

Our setup enabled us to identify effects of small compounds on tumor growth in zebrafish xenografts with Ewing sarcoma, osteosarcoma, glioblastoma, and neuroblastoma cells. Of note, we provide in vivo evidence for efficacy of YAP/TAZ/TEAD inhibition by K-975 against Ewing sarcoma and osteosarcoma xenografts. In addition, we show the benefit of our platform for fast in vivo investigation of newly synthesized compounds, which can then be optimized in in vitro/in vivo iterative cycles. The here reported efficacy of YAP/TAZ/TEAD and Hsp90 inhibition might provide new treatment strategies against Ewing sarcoma and osteosarcoma, most likely then combined with chemotherapy.

Here, we also showcase that such clinically relevant combination treatments, such as ALKi in combination with chemotherapy in neuroblastoma, can be investigated in vivo using our workflow within only one week. This is of particular relevance, since ALKi are the first targeted drugs in first-line treatment of pediatric cancer^34,38^. We observed discrepancies between in vitro and in zebrafish response to ALKi in *ALK*^F1174L^ models. In line with our observation, a loss of response to the ALKi Ceritinib was also observed in vitro upon epithelial-to-mesenchymal transition and in phase I trials in children with ALK-positive malignancies^35,39–42^. In turn, we observed an additive effect of ALKi and chemotherapy in an *ALK*^wt^ xenograft model (SK-N-MM). While we currently do not understand the molecular basis of these differences, the zebrafish xenografts recapitulate aspects of response (and resistance) in patients and might be useful models to study these properties.

We revisited several practical aspects of xenotransplantation including injection site and cell labeling. Although the yolk is a popular injection site for xenotransplantation of tumor cells into zebrafish due to easy accessibility, we observed better tumor formation and growth when targeting the PVS. In addition, tumor cells injected deep into the yolk are harder to image than tumor cells in the rather superficial PVS. Despite a general good persistence of tumor cells of different entities when injected into the PVS, we did also observe specific site requirements for glioblastoma cells. In our hands U-87 MG cells grew better when injected orthotopically into the brain^43^. This may suggest that some zebrafish-derived growth-promoting factors are only present in this particular microenvironment. Thus, orthotopic injection might also be necessary for other tumor entities in order to establish zebrafish xenografts.

To label tumor cells for imaging, CellTracker^TM^ CM-DiI is the most prevalent dye in zebrafish xenograft literature. We compared CellTracker^TM^ CM-DiI with Cell Trace Violet^TM^. While both dyes performed well in labeling tumor cell clusters at the primary injection site in the PVS, Cell Trace Violet^TM^ largely outperformed CellTracker^TM^ CM-DiI in faithfully highlighting disseminated cells. We often detected DiI-labeled particles in the caudal hematopoietic tissue in the absence of actual GFP-expressing tumor cells. As a lipophilic dye, DiI likely also still labels dead cells and cell fragments even after uptake by macrophages and might even get transferred to other cells^11^. The transfer of DiI between living and dead cells was indicated in Kruyt et al.^44^.

To increase the drug screening throughput, we established a robust and automated workflow for image acquisition of xenografted zebrafish, tumor cell detection, and tumor size quantification using a high-content imager (Operetta CLS, PerkinElmer). While we show that it is possible to apply a prescan/rescan strategy to identify zebrafish in regular square bottom imaging plates, we found restricting the area where zebrafish are placed in the well upfront with specific inserts reduces imaging time and increases throughput.

Such a plate format is realized in commercially available ZF plates (Hashimoto). As the thick glass bottom of the original ZF plates used in this study limited their application to low magnification objectives with longer working distances, we also developed 3D-printed inserts, which we successfully used to modify ibidi view plates, but which can also be easily adapted to any desired plate. However, ZF plates are now also available with thinner glass bottoms better suited for higher magnification objectives with shorter working distance.

By using existing analysis modules of the Harmony software (PerkinElmer), we were able to detect tumor cells in zebrafish xenografts in an automated way. This not only tremendously sped up tumor cell detection, but also ruled out biases as compared to manual annotation. Still, automated quantification of tumor size remains a challenge. While volume quantification is the most accurate approach, this strategy is hampered by optical challenges, mainly scattering and absorption by human cells and also acquisition time limitations for recording z-stacks with large numbers of planes for adequate 3D reconstruction. As we typically encountered 3D reconstructions distorted in z-direction leading to overestimation of actual tumor volume, we turned to quantifying the tumor footprint area. This delivered reliable and reproducible results for following size changes of rather spherical tumors. However, this projection approach has obvious limitations should a tumor alter size only along the z-direction. Furthermore, depending on the mode of action of the applied compounds and the time needed to achieve effects, readouts other than tumor area measurements might be necessary.

In summary, we present a setup for high-content imaging-assisted compound screening on zebrafish xenografts for a variety of tumor entities. A checklist with common considerations when applying zebrafish xenografts for drug screening is provided in Supplementary Figure 12. The zebrafish xenograft model together with automated imaging and image analysis as shown here promises to be a powerful system widely applicable for drug testing for human tumor entities bridging the gap between in vitro screening and mouse xenografts. Furthermore, by using patient-derived zebrafish xenografts^4^ this setup can be adapted for personalized medicine approaches to identify patient-tailored drugs and drug combinations within a clinically relevant time span.

## Materials and methods

### Zebrafish strains and husbandry

Zebrafish (*Danio rerio*) were housed under standard conditions^45,46^ according to guidelines of the local authorities (Magistratsabteilung 58) under licenses GZ:565304-2014-6 and GZ:534619-2014-4. For all experiments transparent zebrafish mutants (mitfa^b692/b692^; ednrba^b140/b140^) or transparent zebrafish with labeled vasculature (mitfa^b692/b692^; ednrba^b140/b140^, kdrl:Hsa.HRAS-mCherry^s896tg^) were used. Experiments were performed under license GZ:333989-2020-4.

### Cell culture

GFP-expressing SK-N-MC cells (shSK-E17T^24^, SKshctrl), TC32 (kindly provided by Heinrich Kovar, CCRI, Austria), OS143B (kindly provided by Chrystal Mackall, Stanford University, USA) and Nalm-6 cells were cultured in RPMI 1640 medium with GlutaMAX^TM^ (Gibco, Thermo Fisher Scientific, USA) supplemented with 10% (v/v) FBS and 1% (v/v) Penicillin-Streptomycin (P/S) (10,000 U/ml, Gibco, Thermo Fisher Scientific, USA). shSK-E17T cells particularly were cultured on manually fibronectin-coated plates. Neuroblastoma cell lines, SK-N-BE(2)C-H2B-GFP (kindly provided by Frank Westermann, DKFZ, Germany), SK-N-MM (kindly provided by Nai-Kong Cheung, Memorial Sloan Kettering Cancer Center, US) and a patient-derived culture, STA-NB-8 (established by the authors), were grown in RPMI 1640 medium with GlutaMAX^TM^ supplemented with 10% (v/v) FBS, 80 units/ml penicillin, 80 µg/ml streptomycin (Thermo Fisher Scientific, 15140122, USA), 1 nM sodium pyruvate (Pan-Biotech, P0443100, Germany) and 25 mM Hepes buffer (Pan-Biotech, P0501100, Germany). DsRed expressing HD-MB03 cells were cultured in RPMI + 10% (v/v) FBS + 1% P/S (v/v) supplemented with 1% (v/v) NEAA (100x, #11140050, Gibco, Thermo Fisher Scientific, USA). TagRFP expressing A673-1c^24^ and GFP-expressing U-87 MG cells were expanded in DMEM with GlutaMAX^TM^ (Gibco, Thermo Fisher Scientific, USA) supplemented with 10% (v/v) FBS, 1% (v/v) Penicillin-Streptomycin, A673-1c were further supplemented with 10 µg/ml Blasticidin (InvivoGen, USA) and 50 µg/ml Zeocin (InvivoGen, USA). Primary Ewing sarcoma cells were obtained from a mouse patient-derived xenograft (IC-pPDX-87^47^) (see Table 1).

Mouse PDX experiments of this study were performed in accordance with the recommendations of the European Community (2010/63/UE) for the care and use of laboratory animals. Experimental procedures using patient samples left over after diagnostic procedures were specifically approved by the ethics committee of the Medical University of Vienna (EK1853/2016, EK1216/2018) and Institut Curie CEEA-IC #118 (Authorization APAFIS#11206-2017090816044613-v2 given by National Authority) in compliance with the international guidelines. Written informed consent by patients or their legal representatives allowing generation of PDX models was obtained.

Tumor pieces were dissociated according to Stewart et al. and IC-pPDX-87 cells were short-term cultured (<5 passages) in DMEM/F-12 with GlutaMAX^TM^ (Gibco, Thermo Fisher Scientific, USA) supplemented with 1% (v/v) B-27 (50X, Gibco, Thermo Fisher Scientific, USA) and 1% (v/v) P/S^48^. HEK293T cells were used only for lentiviral particle production and were grown in Dulbecco’s Modified Eagle’s Medium (DMEM) supplemented with 10% (v/v) FBS, 2 mM L-glutamine, 100 units/ml penicillin, and 100 μg/ml streptomycin. All cells were kept in an atmosphere with 5% CO_2_ at 37 °C. At 90% confluence cells were passaged using Accutase (Pan Biotech, Germany).

### Viability assay at lower temperature

Neuroblastoma cells were seeded in T-25 flasks (750,000 cells/flask) and incubated for three days at 34 °C or 37 °C. Then the cells were harvested and were manually counted using a hemocytometer.

### In vitro drug dose–response assays

Cells were seeded in 96-well white opaque plates (Cat. No. 6005680, PerkinElmer, USA,) and incubated at 37 °C for 24 h. They were then treated with increasing concentrations of Ceritinib or Temozolomide and 72 h later cell viability was measured with the CellTiter-Glo® Luminescent Cell Viability Assay (Cat. No. G7573, Promega, USA)) following the manufacturer’s instructions.

### Lentiviral transfection of cell lines

The neuroblastoma cell line SK-N-MM and patient-derived culture STA-NB-8 with stable GFP expression were generated by lentiviral spinfection following the appropriate biological safety regulations. To deliver plasmids to generate the lentiviral particles encoding the GFP protein, lipofectamine 3000 (ThermoFisher Scientific, USA) was used according to the manufacturer´s protocol. 10 μg of the plenti plasmid pCLS-CV^49^ encoding the GFP protein was incubated with 5.4 μg of packaging plasmids (pMDLg/pRRE #11251, pRSV-Rev #11253, pMD2.g #12259, all from Addgene, USA) in 1500 μl Opti-MEM I (ThermoFisher Scientific, USA) with 30 µl P3000 enhancer (ThermoFisher Scientific, USA). The mixture was vortexed for 10 s. In a separate mix, 46 μl of lipofectamine 3000 (ThermoFisher Scientific, USA) was added to 1500 μl Opti-MEM I and was gently added to the first mixture followed by 4 min incubation at room temperature. The transfection mix was added drop-wise to a 10-cm dish of ~70% confluent HEK293T cells. 6 h post-transfection, the medium was replaced with lentiviral packaging medium (Opti-MEM I, 5% FCS, 200 µM sodium pyruvate). 24 h later, the lentiviral medium was collected and filtered through 0.45 µm filters. Target cells (~60% confluent) were spinfected with the optimized titer of the lentiviral medium by centrifugation at 800 × *g* for 45 min at 32 °C. Spinfected cells were recovered for one day in fresh medium and GFP expression was confirmed by flow cytometry.

### Preparation of cells for transplantation

To prepare fluorescently labeled cells for xenotransplantation cells were detached from the culture dish with Accutase (PAN-Biotech, Germany). After a centrifugation step (5 min, 500 × *g*, 4 °C) cells were taken up in PBS supplemented with 2% FBS and put through a 35 µm cell strainer (5 ml polystyrene round bottom tubes with cell strainer cap, Corning, USA) to disperse cell clumps. Cell number was determined using a Coulter Counter (Z2, Beckman Coulter, USA). Cells were then centrifuged, resuspended to a concentration of 100 cells/nl PBS and kept on ice until transplantation.

### CM-DiI labeling

To label cells with CellTracker^TM^ CM-DiI (Invitrogen, Thermo Fisher Scientific, USA) cells were harvested by incubation with protease. Subsequent to a regular counting and centrifugation routine, cell number was adjusted to a concentration of 1 × 10^6^ cells/ml in serum-free medium. For staining CM-DiI was mixed in at a concentration of 2 µg/ml. Cells were incubated with CM-DiI for 5 min at 37 °C in the dark, followed by 15 min on ice still in the dark, then washed again twice with RPMI supplemented with 2% FBS. Pelleted cells were taken up in PBS supplemented with 2% FBS. MgCl_2_ was added to a final concentration of 2 mM as well as DNase I to a final concentration of 50 µg/ml for 20 min incubation at 37 °C. After two PBS/2% FBS washing steps, cells were put through a 35 µm cell strainer and total cell number was assessed. Cells were then centrifuged again, resuspended to a concentration of 100 cells/nl in PBS and kept on ice until transplantation.

### CellTrace^TM^ Violet labeling

To label cells with CellTrace^TM^ Violet (Invitrogen, Thermo Fisher Scientific, USA) cells were harvested with Accutase and cell numbers adjusted to a concentration of 1 × 10^6^ cells/ml in PBS. CellTrace^TM^ Violet was mixed into a final concentration of 5 µM. Cells were incubated with CellTrace^TM^ Violet for 10 min at 37 °C in the dark. 5 volumes of medium supplemented with 10% FBS were added, and the suspension was incubated for 5 min. After centrifugation (5 min, 500 g, 4 °C) cells were resuspended in fresh medium supplemented with 10% FBS and incubated for another 10 min in the dark. Finally, sticky cells were separated by means of a 35 µm cell strainer and then counted. Cells were centrifuged again, resuspended to a concentration of 100 cells/nl in PBS and kept on ice until transplantation.

### Xenotransplantation

mitfa^b692/b692^; ednrba^b140/b140^ embryos were raised until 2 days post fertilization (dpf) at 28 °C, dechorionated and anesthetized with 1x Tricaine (0.16 g/l Tricaine (Cat No. E1052110G, Sigma-Aldrich Chemie GmbH, Germany), adjusted to pH 7 with 1 M Tris pH 9.5, in E3). To facilitate the transplantation process larvae were aligned on a petri dish lid coated with a solidified 2% agarose solution as described previously^16^.

The borosilicate glass capillaries without filament (GB100T-8P, Science Products GmbH, Germany) for injection of tumor cells were pulled with a needle puller (P-97, Sutter Instruments, USA). Needles were loaded with ~5 µl of cell suspension, mounted onto a micromanipulator (M3301R, World Precision Instruments Inc., Germany) and connected to a microinjector (FemtoJet 4i, Eppendorf, Germany). Approximately 200–400 cells were transplanted into the perivitelline space (PVS), the optic tectum or into circulation of zebrafish larvae. Following an inspection under the microscope larvae carrying tumor cells only at/ in the respective injection site at 2 h post injection (hpi) were selected and subsequently maintained at approx. 34 °C.

### Whole mount immunostaining

Xenotransplanted larvae were fixed in 4% PFA (Electron Microscopy Sciences, USA) in PBS overnight at 4 °C. Larvae were washed once with PBS and then gradually transferred to 100% MetOH for storage at −20 °C. For immunostaining, larvae were gradually transferred back to PBS. After washing once with PBSX (PBS with 0.1% Triton X-100) and washing once with plenty distilled water, larvae were incubated in acetone for 7 min at −20 °C. Larvae were blocked for 1 h in PBDX (PBS with 0.1% Triton X-100, 0.1 g/l BSA, 0.1% DMSO) supplemented with 15 µl goat serum (GS) (normal donor herd, Sigma-Aldrich, USA)/ml PBDX. Dilutions of primary antibodies against Ki-67 (1:400) ((8D5) mouse primary mAb #9449, Cell signaling Technology, USA) and Cleaved Caspase 3 (1:100) ((D175) primary antibody, Cell Signaling Technology, USA) in PBDX + GS were prepared. Antibody incubation routine for larvae was in primary antibody solution overnight at 4 °C, followed by 4x washing in PBDX for 30 min, then secondary antibodies Alexa 568 anti-mouse (A-11019, Invitrogen, USA) or Alexa 568 anti-rabbit (A-21069, Invitrogen, USA) diluted 1:500 in PBDX + GS for 1 h. From this point on all steps were carried out ensuring that samples are kept in the dark. Larvae were washed twice with PBDX and 1x with PBS, followed by an incubation in 4% PFA for 5 min. Larvae were washed 3 times with PBS, transferred to Dako Fluorescence Mounting Medium (Dako, Agilent, USA), and stored at 4 °C.

### Imaging

Fluorescence images of anesthetized larvae were acquired using an Axio Zoom.V16 fluorescence stereo zoom microscope with an Axiocam 503 color camera from Zeiss (Zeiss, Germany) using the image ZEN software (Zeiss, Germany). Fixed and immunostained larvae were embedded in 1.2% ultra-low gelling agarose (Cat. No. A2576-25G, Sigma-Aldrich Chemie GmbH, Germany) in a glass bottom imaging dish (D35-14-1.5-NJ, Cellvis, USA) as previously described^50^. Imaging was performed using a SP8 X WLL confocal microscope system and the LASX software (Leica, Germany).

### Quantification of immunofluorescence

Confocal images of immunostained xenotransplanted tumors were quantified using the ImageJ software (version 1.53c). Images were split into green and red channels and threshold was set manually to detect the fluorescence area. Fluorescence area was determined for whole tumor versus Ki67- and cleaved Caspase 3-signals and percentages were calculated.

### Automated imaging and quantification

For automated imaging at 1 and 3 dpi larvae were anesthetized in 1x Tricaine and embedded in either an ibidi µ-plate with glass bottom (Cat. No. 89627, Ibidi, Germany) or a 96-well ZF plate (Cat. No. HDK-ZFA 101, Hashimoto Electronic Industry Co, Japan). Larvae were transferred with minimal residual volume into 0.5% ultra-low gelling agarose/1x Tricaine (Cat. No. A2576-25G, Sigma-Aldrich Chemie GmbH, Germany) as a compromise concentration between mechanical support for proper orientation of larvae and easy retrieval after imaging. Using large orifice tips (Cat. No. E1011-8400, Starlab, USA) xenografted larvae were pipetted into the respective well in a volume of 200 µl. GELoadertips (Cat. No. 0030001.222, Eppendorf, Germany) were used to gently orientate the larvae into the correct position (head left) towards the bottom of the well. After imaging, large orifice tips were used again to pipette larvae out of the imaging plate into a drug treatment plate.

The Operetta CLS high-content imager (PerkinElmer, USA) was used for image acquisition in confocal mode and with defined settings: 5x air objective, Brightfield (40 ms, 10%), GFP (excitation: 460–490 nm at 100%, emission: 500–550 nm for 400 ms), DiI and tagRFP (excitation: 530–560 nm at 100%, emission: 570–650 nm for 400 ms), CellTrace Violet (excitation: 390–420 nm at 100%, emission: 430–500 nm for 600 ms). Larvae were imaged in 2 fields of view (covers whole larvae), with 23 planes with a distance of 25 µm per field (approx. optimal for this objective according to manufacturer). Tumor size was quantified with the Harmony Software 4.9 (PerkinElmer, USA). The area of the tumor projected along the z-axis onto the x-y-plane (“footprint area”) was used for further analysis of tumor growth form 1 dpi to 3 dpi. In more detail, the slot area per well was defined in the brightfield channel by threshold against the dark well walls to limit the area of analysis. Next, the fluorescence signal from tumor cells was detected by applying the lowest possible intensity threshold (vs infinite highest intensity) at a scale suitable for all samples of the plate in the 3D analysis mode. In case tumor cells separated from the primary tumor, a size threshold was added to select for the largest fluorescent bolus, which is typically the primary tumor. Aside from size, Harmony software can delineate position of detected fluorescence areas in X and Y. This function can be used to find fluorescence signal in a specific area. In case of disturbing autofluorescence e.g., from the yolk it is advised to restrict the analysis to a certain region this way.

For detection of cells in circulation (Nalm-6 or migrated cells) either a spot detection algorithm of the Harmony software was used or the fluorescent area outside of the primary tumor in maximum projection was quantified.

### 3D-printed inserts for imaging

Developing and processing of the inserts and stamps was conducted in three steps. Inserts and stamps were designed using Autodesk Inventor professional 2020. The design of the stamps was adapted from Wittbrodt et al.^25^. Inserts and the stamps were printed with a Hot Lithography 3D printer (Caligma, Cubicure GmbH, Vienna) using the photopolymer Cubicure Evolution (Cubicure GmbH, Austria). The printer polymerized the material layerwise using a UV-laser (wavelength: 375 nm). The thickness of the individual layers was 100 µm. Printed parts were cleaned iteratively using an acetate-based solvent and an ultrasonic bath. Post curing was conducted at 70 °C. Inserts fit into Ibidi 96 Well Black µ-Plates (Cat. No. 89621, uncoated, Ibidi, Germany).

To form stable grooves with the stamps, 300 µl 2% (w/v) ultra-low gelling agarose (Sigma-Aldrich Chemie GmbH, Germany) was poured into each of the wells of an ibidi 96-well plate, the stamps were instantly inserted and for a few minutes left there while in the refrigerator. When agarose was solidified, stamps were carefully removed.

### Determination of “No observed effect concentration” (NOEC)/compound treatment

YK-4-279 (Cat. No. HY-14507, MedChemExpress, Sweden), K-975 (Cat. No. HY-138565, MedChemExpress, Sweden), A-1331852 (Cat. No. HY-19741, MedChemExpress, Sweden), 17-DMAG (Alvespimycin) (Cat. No. HY-10389, MedChemExpress, Sweden) and freshly synthesized Hsp90 inhibitors were dissolved in DMSO to a stock concentration of 10 mM. Temozolomide (Cat. No. HY-17364, MedChemExpress, Sweden) and Ceritinib (Cat. No. HY-15656A) were dissolved in ddH_2_O to a stock concentration of 10 mM. Not all compounds can be solved in water/E3 or DMSO. For alternative solvents and applicable concentrations in zebrafish we suggest the following article: Maes et al., *Plos One* 2012^51^. Selection of the proper solvent should be based on informed decision considering solubility of compounds of interest as well as tolerance of larvae.

As starting point for NOEC determination, we typically investigate concentrations around 10x of in vitro IC50s. This matches a reported 10 % uptake by zebrafish larvae of compounds delivered to the water (determined for paracetamol)^52^.

Here, to define the highest tolerated concentration at approx. 34 °C various dilutions in fish E3 media of YK-4-279 (2,5 µM, 5 µM, 10 µM, and 20 µM), K-975 (1 µM, 2 µM, 4 µM and 8 µM),Temozolomide (0,5 mM, 1 mM, 2 mM and 5 mM) and Ceritinib (1 µM, 2 µM, 5 µM and 15 µM) were tested. Treatment of 10–12 pigment mutant zebrafish larvae in regular 12-well multi-well plates started at 3dpf. Impact on larval health and survival was assessed by inspection on a brightfield microscope at the end of a 48 h treatment.

For drug screening experiments, cells were transplanted into the PVS of 2 dpf old zebrafish larvae as described above. Tumor cells were allowed to grow for 24 h to form a compact tumor mass at the injection site. After recording reference images of the xenotransplanted larvae at 1 dpi in the Operetta CLS, they were transferred to cell culture plates containing fresh E3 embryo medium and accordingly compound in indicated concentrations for an incubation time of 48 h. The applied compounds did not need refreshment during treatment, as supernatant was still active at the end of the assay (tested on fresh xenografts or during in vitro assays), but this needs consideration during experiment planning. Dishes containing light sensitive compounds were covered with light protection. For easier pipetting compound treatments were conducted in 48-well or 24-well format (single larvae per well). At 3 dpi, larvae were imaged again post-treatment on the Operetta CLS.

### Chemistry

The reagents and solvents for the synthesis were purchased from Enamine Ltd (Kyiv, Ukraine), Apollo Scientific Ltd (Stockport, UK) and Sigma-Aldrich (St. Louis, MO, USA) and were used without further purification. Analytical thin-layer chromatography was performed on silica gel aluminum sheets (0.20 mm; 60 F254; Merck, Darmstadt, Germany) to monitor the progress of the reactions, while flash column chromatography, used to purify the compounds, was performed on silica gel 60 (particle size, 230–400 mesh). A 400 MHz NMR spectrometer Bruker Advance 3 (Bruker, Billerica, MA, USA) was used to record the ^1^H and ^13^C NMR spectra. The splitting patterns of the peaks were designated as s for singlet, d for doublet, dd for doublet of doublet, ddd for double of doublet of doublet, and m for multiplet. The mass spectra of the prepared compounds were recorded using Expression CMS^L^ mass spectrometer (Advion Inc., Ithaca, NY, USA), while Exactive Plus Orbitrap mass spectrometer (Thermo Scientific Inc., Waltham, MA, USA) was used to record the high-resolution mass spectra (HRMS) of the final product. The purity of TSF-15 was determined by analytical reversed-phase UHPLC on the Thermo Scientific Dionex UltiMate 3000 UHPLC modular system (Thermo Fisher Scientific) equipped with a photodiode array detector set to 254 nm. A Waters Acquity UPLC^®^ HSS C18 SB column (1.8 μm, 2.1 mm × 50 mm) thermostatted at 40 °C was used. The mobile phase consisted of 0.1% TFA in H_2_O (A) and MeCN (B), using the following gradient: 95% A to 5% A in 7 min, then 95% B for 1 min, with a flow rate of 0.3 mL/min and an injection volume of 2.5 µL. 17-DMAG was purchased from MedChemExpress. Synthesis of TSF-15 is presented in Supplementary Figure 9.

Synthesis of (*S*)-*N*-(2-amino-4,5,6,7-tetrahydrobenzo[*d*]thiazol-6-yl)-3,4-dichlorobenzamide (TSF-2). 3,4-Dichlorobenzoic acid (3.39 g, 17.8 mmol) was dissolved in *N*,*N*-dimethylformamide (DMF, 15 mL). EDC (3.31 g, 21.3 mmol), HOBt (3.53 g, 23.1 mmol) and NMM (3.86 mL, 35.5 mmol) were added on an ice bath. The reaction mixture was stirred for 15 min and then (*S*)-4,5,6,7-tetrahydrobenzo[*d*]thiazole-2,6-diamine (3.00 g, 17.8 mmol) was added. The reaction mixture was stirred overnight at room temperature. The solvent was evaporated under reduced pressure. EtOAc (50 mL) and NaHCO_3_ (50 mL) were added to the residue and a precipitate was filtered off. The precipitate was then washed with MeOH to yield a clean product. Yield: 56% (3.400 g); white solid; R_f_ (DCM:MeOH = 9:1) = 0.37; ^1^H NMR (400 MHz, DMSO-*d*_*6*_): δ = 8.64 (d, *J* = 7.6 Hz, 1H), 8.10 (d, *J* = 2,1 Hz, 1H), 7.84 (dd, *J*_*1*_ = 8.4 Hz, *J*_*2*_ = 2.1 Hz, 1H), 7.76 (d, *J* = 8.4 Hz, 1H), 6.68 (s, 2H), 4.23–4.13 (m, 1H), 2.86–2.76 (m, 1H), 2.00–1.91 (m, 1H), 1.88–1,75 (m, 1H) ppm, the signals of the remaining protons are overlapped with the signal for DMSO-*d*_*6*_ (Supplementary Figure 10a); MS (ESI^+^) for C_14_H_14_Cl_2_N_3_OS *m/z*: 341.9 [M + H]^+^.

Synthesis of *tert*-butyl (3-(((*S*)-6-(3,4-dichlorobenzamido)-4,5,6,7-tetrahydrobenzo[*d*]thiazol-2-yl)amino)-3-oxo-1-phenylpropyl)carbamate (TSF-10). 3-((*tert*-Butoxycarbonyl)amino)-3-phenylpropanoic acid (113 mg, 0.585 mmol) was dissolved in DMF. EDC (109 mg, 0.701 mmol), HOBt (116 mg, 0.760 mmol) and NMM (0.204 mL, 1.17 mmol) were added on an ice bath. The reaction mixture was stirred for 15 min and then TSF-2 (200 mg, 0.585 mmol) was added. The reaction mixture was stirred overnight at room temperature. The solvent was evaporated under reduced pressure. The residue was taken up in EtOAc (50 mL) and the organic phase was washed with 1% citric acid (2 × 50 mL), saturated NaHCO_3_ (2 × 50 mL), brine (50 mL) and dried over Na_2_SO_4_. The solvent was evaporated and the residue was further purified by flash column chromatography using DCM:MeOH = 20:1 as the mobile phase. Yield: 40% (138 mg); light yellow solid; R_f_ (DCM:MeOH = 9:1) = 0.43; ^1^H NMR (400 MHz, DMSO-*d*_*6*_) δ 11.92 (s, 1H), 8.68 (d, *J* = 7.5 Hz, 1H), 8.11 (d, *J* = 2.0 Hz, 1H), 7.85 (ddd, *J*_*1*_ = 8.5 Hz, *J*_*2*_ = 2.0 Hz, *J*_*3*_ = 0.8 Hz, 1H), 7.77 (dd, *J*_*1*_ = 8.5 Hz, *J*_*2*_ = 0.8 Hz, 1H), 7.51 (dd, *J*_*1*_ = 8.8 Hz, *J*_*2*_ = 4.0 Hz, 1H), 7.36–7.27 (m, 4H), 7.26–7.19 (m, 1H), 5.03 (q, *J* = 8.0 Hz, 1H), 4.22 (s, 1H), 3.00 (dd, *J*_*1*_ = 15.7 Hz, *J*_*2*_ = 5.3 Hz, 1H), 2.83–2.77 (m, 2H), 2.72–2.63 (m, 2H), 2.07–1.97 (m, 2H), 1.95 – 1.81 (m, 1H), 1.39–1.18 (m, 9H) ppm (Supplementary Figure 10b); MS (ESI^+^) C_28_H_31_Cl_2_N_4_O_4_S *m/z*: 589.8 [M-H]^+^.

Synthesis of 3-(((*S*)-6-(3,4-dichlorobenzamido)-4,5,6,7-tetrahydrobenzo[*d*]thiazol-2-yl)amino)-3-oxo-1-phenylpropan-1-aminium chloride (TSF-15). To a solution of compound TSF-10 (60 mg, 0.102 mmol) in 1,4-dioxane (20 mL) 4 M HCl in 1,4-dioxane (0.76 mL, 3.05 mmol) was added and the mixture was stirred overnight at room temperature. The solvent was evaporated under reduced pressure and the solid residue was washed with 1,4-dioxane to yield a clean product. Yield: 84% (45 mg); white solid; R_f_ (DKM:MeOH = 9:1) = 0.0; ^1^H NMR (400 MHz, DMSO-*d*_*6*_) δ 12.16 (s, 1H), 8.69 (dd, *J*_*1*_ = 7.6 Hz, *J*_*2*_ = 3.4 Hz, 1H), 8.50 (s, 3H), 8.10 (t, *J* = 1.7 Hz, 1H), 7.84 (ddd, *J*_*1*_ = 8.4 Hz, *J*_*2*_ = 2.1 Hz, *J*_*3*_ = 1.3 Hz, 1H), 7.77 (dd, *J*_*1*_ = 8.4 Hz, *J*_*2*_ = 1.3 Hz, 1H), 7.54–7.46 (m, 2H), 7.47–7.34 (m, 3H), 4.76–4.64 (m, 1H), 4.26–4.14 (m, 1H), 3.19–3.07 (m, 2H), 3.03–2.95 (m, 1H), 2.70–2.64 (m, 3H), 2.06–1.98 (m, 1H), 1.94–1.81 (m, 1H) ppm (Supplementary Figure 10c); ^13^C NMR (101 MHz, DMSO-*d*_6_) δ 167.00, 163.63, 154.97, 143.38, 136.86, 136.84, 134.81, 133.96, 131.16, 130.64, 129.29, 128.81, 128.70, 127.79, 127.61, 127.59, 119.61, 50.96, 46.17, 46.13, 28.41, 28.09, 24.68 ppm (Supplementary Figure 10d); HRMS (ESI^+^) calcd. for C_23_H_24_Cl_2_N_4_O_2_S [M + H]^+^: 489.0913, found: 489.0907; UPLC: t_r_: 4.060 min (98.08% at 254 nm and 96.77% at 220 nm) (Supplementary Figure 10e, f).

### Hsp90 C-terminal domain time-resolved fluorescence resonance energy transfer (TR-FRET) assay

Hsp90α C-terminal domain TR-FRET kit was purchased from BPS Bioscience (San Diego, USA) and assay was carried out according to manufacturer’s protocol. Sample contained terbium-labeled donor, dye-labeled acceptor, Hsp90α C-terminal domain, PPID and TSF-15 or novobiocin (known Hsp90 CTD inhibitor) or 17-DMAG (known Hsp90 N-terminal domain inhibitor). Positive control contained no inhibitor, negative control did not contain target protein PPID. Samples were incubated for 2 h, then protein–protein interaction between Hsp90 C-terminal domain and PPID was assayed by measuring TR-FRET using Tecan’s Spark Multimode Microplate reader (Tecan Trading AG, Switzerland). Each sample was performed in triplicate. Percentage of C-terminal domain activity was calculated using the following formula %Activity = 100 × (FRET_sample_ – FRET_negative control_)/(FRET_positive control_ – FRET_negative control_), where FRET value is ratio between dye-acceptor emission and Tb-donor emission. Statistical analysis was performed using one-way ANOVA post hoc Dunnett’s test (Supplementary Figure 11a).

### MTS assay

The antiproliferative activity of TSF-15 against an Ewing sarcoma cell line SK-N-MC was evaluated, using an MTS (Promega, Madison, WI, USA) assay, according to the manufacturer’s instructions. The cells were cultured in RPMI medium with HEPES (Sigma-Aldrich, St. Louis, MO, USA), which was supplemented with 10% fetal bovine serum (Gibco, Thermo Fisher Scientific, Waltham, MA, USA), 100 U/mL penicillin (Sigma-Aldrich, St. Louis, MO, USA), 100 µg/mL streptomycin (Sigma-Aldrich, St. Louis, MO, USA), and 2 mM L-glutamine (Sigma-Aldrich, St. Louis, MO, USA). The cells were incubated in a 5% CO_2_ atmosphere at 37 °C. For testing the cells were plated in 96-well plates at a density of 2000 cells/well. Afterwards, the cells were incubated for 24 h, and then treated with TSF-15 and vehicle control (0.5% DMSO). Afterward, the cells were incubated with the compound for 72 h and then CellTiter96 Aqueous One Solution Reagent (10 µL; Promega, Madison, WI, USA) was added to each well. The cells were incubated for an additional 3 h and then the absorbance was measured using a microplate reader (Synergy 4 Hybrid; BioTek, Winooski, VT, USA). Independent experiments were repeated two times, each performed in triplicate. The statistically significant differences (*p* < 0.05) were calculated between the treated groups and DMSO, using two-tailed Welch’s t-tests. GraphPad Prism 8.0 (San Diego, CA, USA) was used to determine the IC_50_ value of TSF-15, which represents the concentration at which the compound produced a half-maximal response (given as means from the three independent measurements) (Supplementary Figure 11b). 17-DMAG showed antiproliferative activity in SK-N-MC cell line with an IC_50_ of 0.01 ± 0.007 µM^53^.

### Statistical analysis

Statistical analysis was performed in Prism 8 (Version 8.3.0, Graphpad, USA). Distribution of data was tested with a normality test (D’Agostino & Pearson). Based on data distribution either parametric tests (student’s t-test or ANOVA) or non-parametric tests (Mann–Whitney test or Kruskal–Wallis test) were performed. The applied method is indicated in the figure legends.

### Reporting summary

Further information on research design is available in the Nature Research Reporting Summary linked to this article.

## Supplementary information

Supplementary Figures

REPORTING SUMMARY

## Data Availability

The datasets generated during and/or analyzed during the current study are available from the corresponding author on reasonable request.

## Code availability

CAD files for 3D-printed inserts are available from the following repository: 10.48436/7qfnf-qpy18.
