# Supplementary material for: Refined high-content imaging-based phenotypic drug screening in zebrafish xenografts
Source: NPJ Precis Oncol. 2023 May 18;7:44. doi: 10.1038/s41698-023-00386-9 (PMC10195872; doi:10.1038/s41698-023-00386-9)
Supplement: Supplementary file 1 — Supplementary Figures [file 41698_2023_386_MOESM1_ESM.pdf]

Supplementary Figures

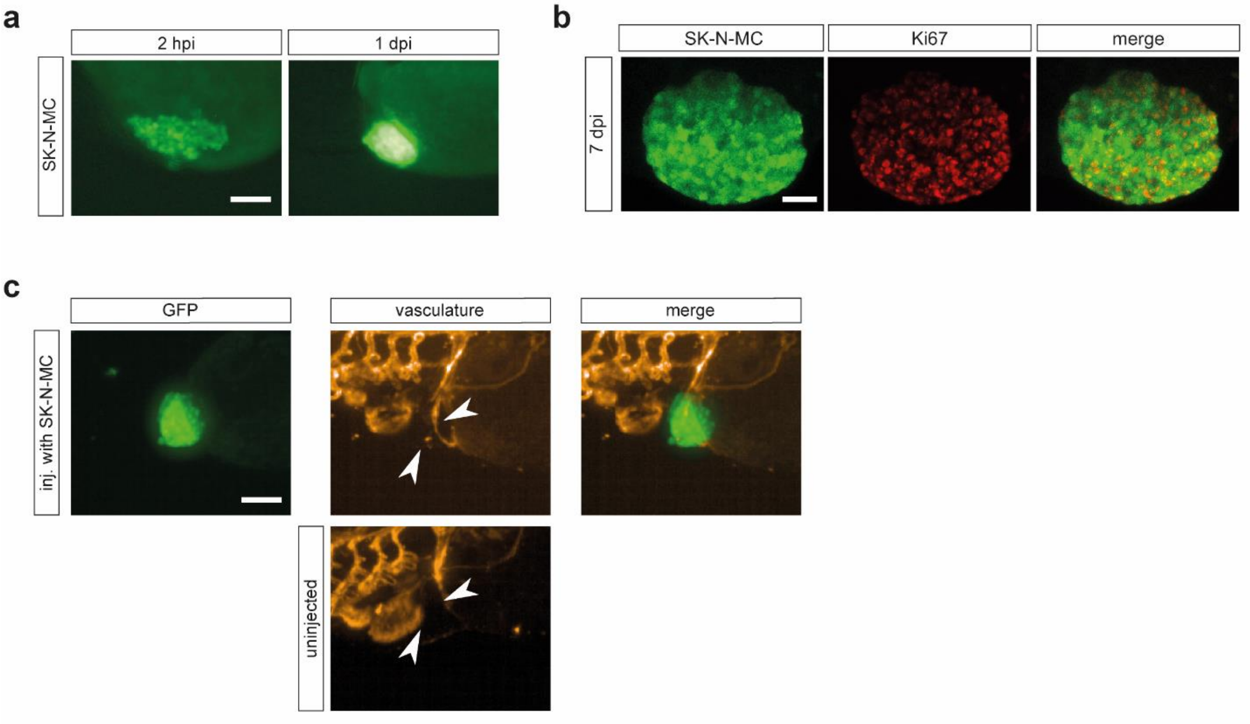

Supplementary Figure 1: Proliferation and vascularization of Ewing sarcoma cells (ShSK-E17T) in xenografts: a) Remodelling of Ewing sarcoma cells from a loose mass at 2 hpi to a solid tumor at 1 dpi. Scale bar is 100 µm. b) Transplanted embryo was fixed at 7 dpi and immunostained for Ki67. Scale bar is 50 µm. c) Ewing sarcoma cells were transplanted into transgenic Tg(kdrl:Hsa.HRAS-mCherry^{s896}) zebrafish embryos to show interaction of tumor cells with zebrafish vasculature at 3 dpi. Uninjected Tg(kdrl:Hsa.HRAS-mCherry^{s896})larvae were imaged as a control. Scale bar is 100 µm.

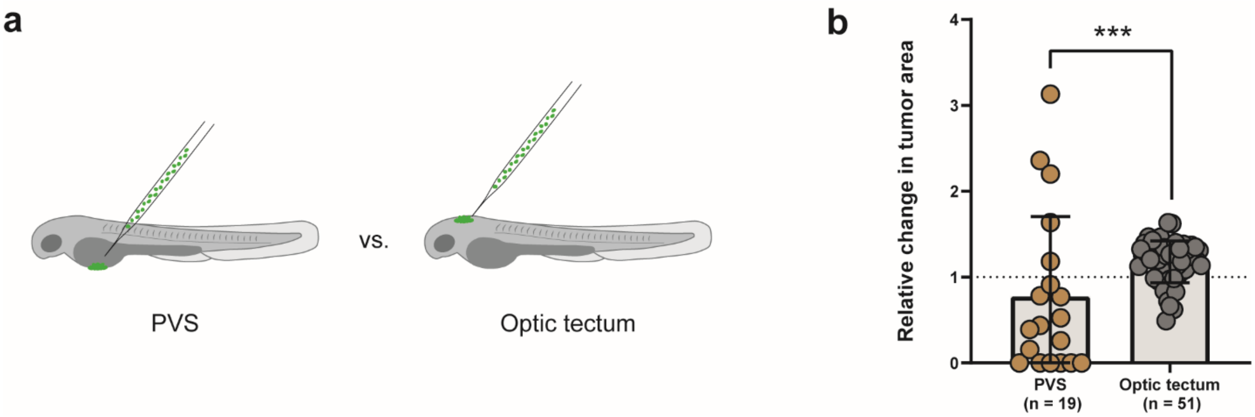

Supplementary Figure 2: Orthotopic transplantation of U-87 MG cells: a) U-87 MG glioblastoma cells were transplanted into 2 dpf old zebrafish larvae either at the PVS or the optical tectum. b) Dot plot shows relative change in tumor size for transplantation into the PVS (n = 19) or into the optic tectum (n = 51). Statistical analysis was performed with a Mann-Whitney test (*** p=0.001). Error bars represent SD.

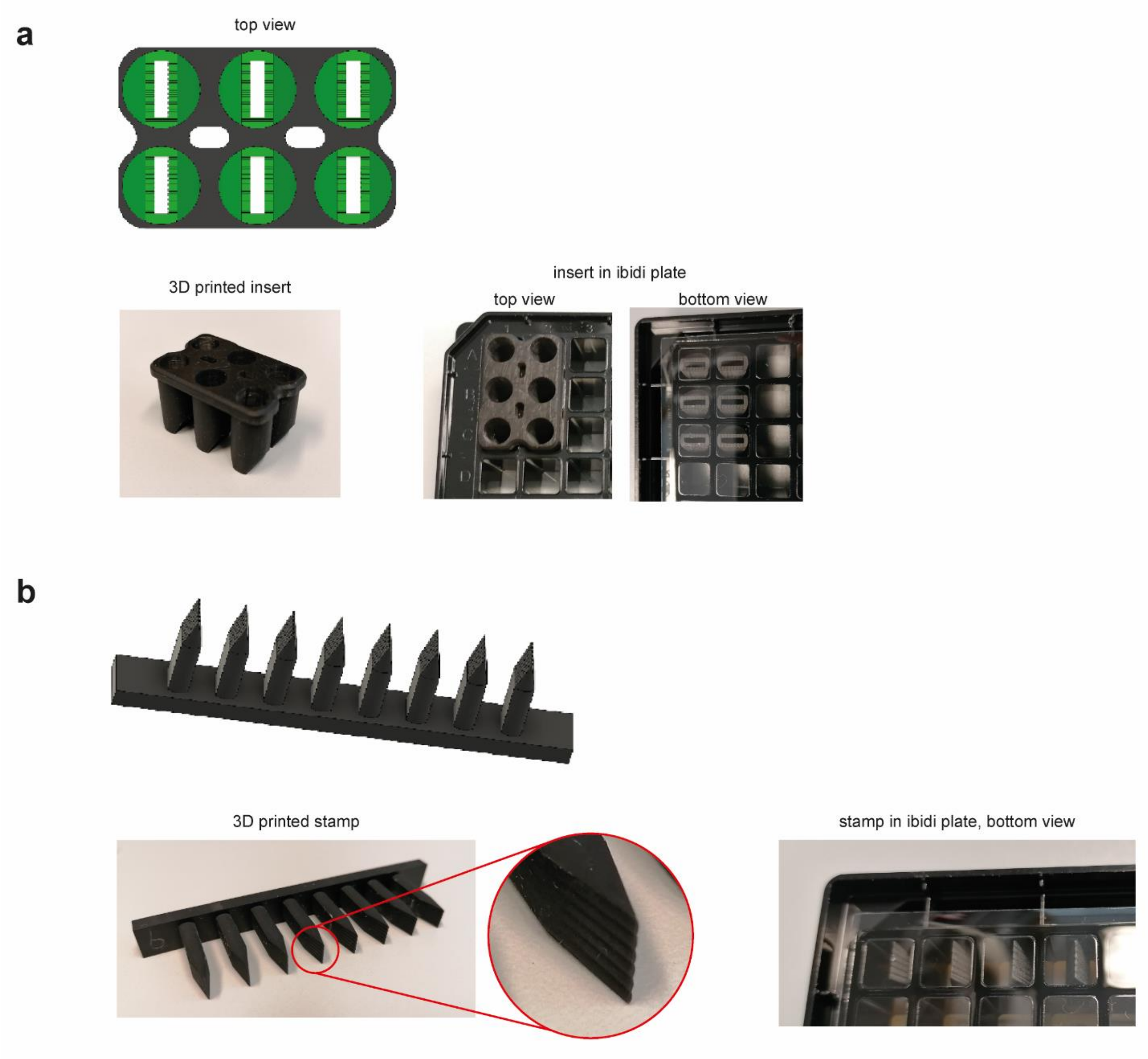

Supplementary Figure 3: 3D-printed inserts & stamps for ibidi plates: a) Model of the inserts from different views, image of printed inserts, and inserts placed in ibidi plate, b) Model of stamps with a wave tip to ensure the placement of the zebrafish, printed and post processed stamps and stamps placed in the ibidi plate. Size in mm.

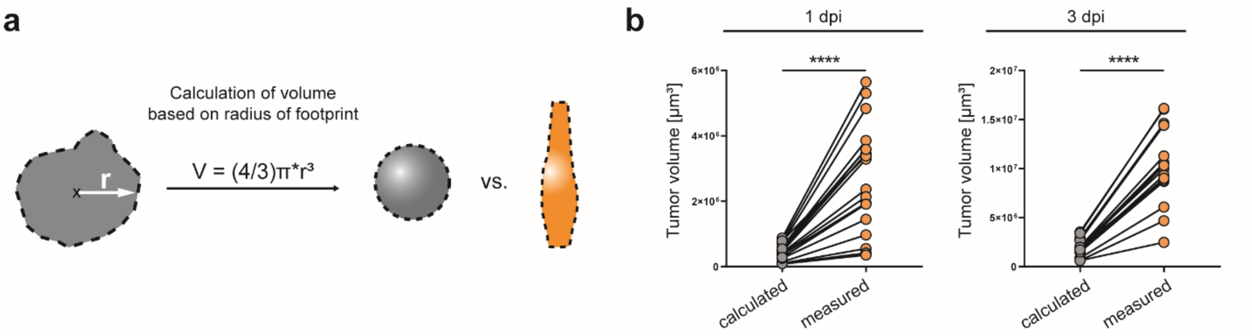

Supplementary Figure 4: Comparison of hypothetical tumor radius with volume measurement: a) Calculation of the radius based on the footprint of the tumor enables the calculation of a hypothetical tumor volume that can be compared to the measured tumor volumes. b) Dot plot show the comparison between calculated tumor volumes (grey) and measured tumor volumes (orange) at 1 dpi and 3 dpi. Statistical analysis was performed with a paired t-test (**** $p<0.0001$).

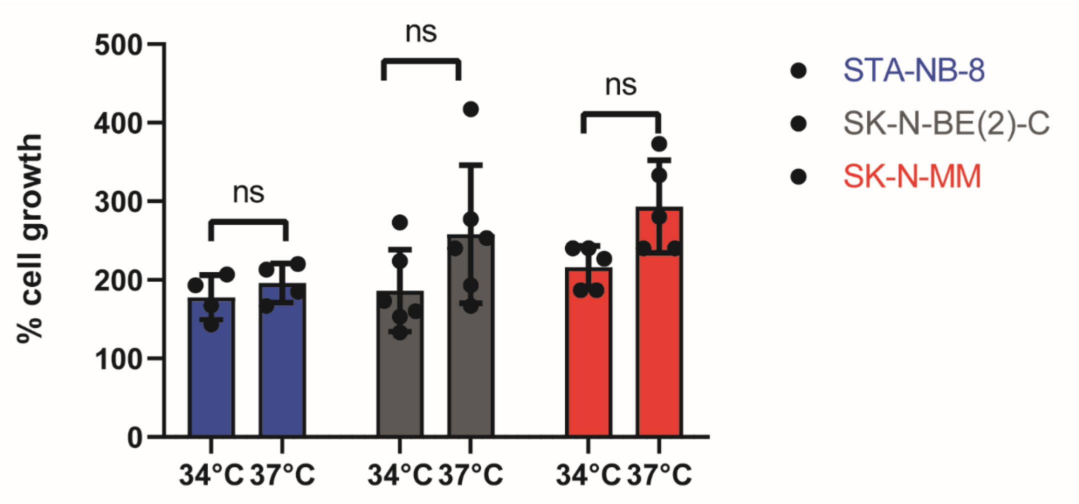

Supplementary Figure 5: Comparison of cell growth of neuroblastoma cell lines at 37°C vs. 34°C: Neuroblastoma cells were seeded and incubated for 3 days at 34°C or 37°C. Cell viability was calculated as the percentage of the total number of viable cells to the number of cells seeded. 3 biological replicates were plotted. Statistical analysis was performed with 2-way ANOVA with Bonferroni corrections. ns: not significant. Error bars represent standard deviation.

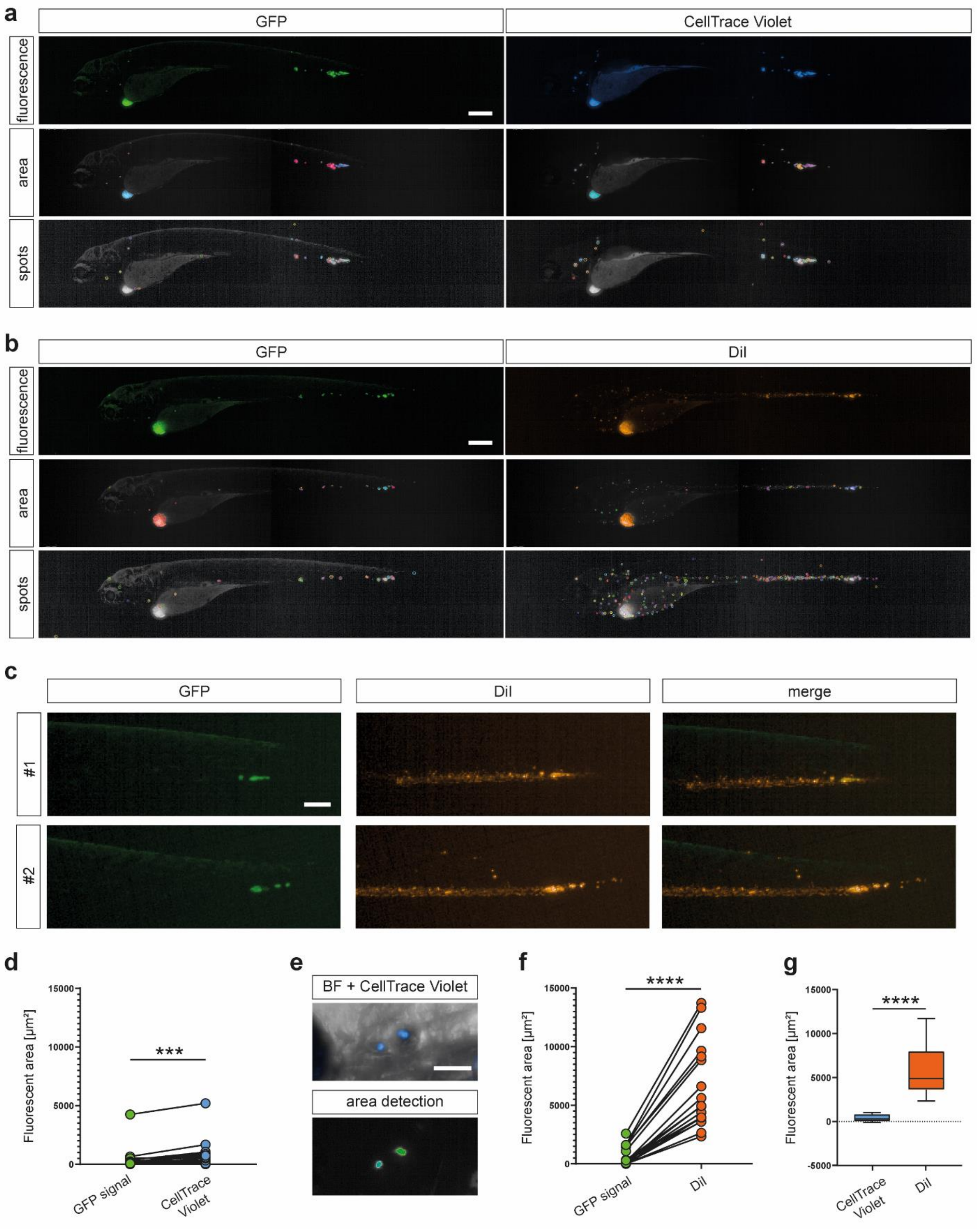

Supplementary Figure 6: Comparison between GFP and DiI signals in the tail: a-b) GFP-labeled SK-N-MC cells (Skshctrl) were counter-labeled with CellTracker CM-DiI and imaged at 3 dpi with the Operetta CLS. Area detection and spot count analysis was used to determine disseminated cells. Scale bars are 250 µm. c) Two examples showing excessive DiI staining in the tail region without many GFP-positive cells being present. Scale bar is 100 µm. d&f)

Fluorescent area for GFP, CellTrace Violet and DiI (excl. primary tumor) was determined with the Harmony software. Statistical analysis was performed with a paired t-test (*** $p=0.0003$, **** $p<0.0001$). e) Detection of otoliths in CellTrace Violet channel. Scale bar is 100 µm. g) Difference of CellTrace Violet- or DiI-area to GFP was calculated and plotted with a Tukeys box plot. Line represents the median value, box spans 25th to 75th percentile, and whiskers span 5th to 95th percentile. Statistical analysis was performed with a Mann-Whitney test (**** $p<0.0001$).

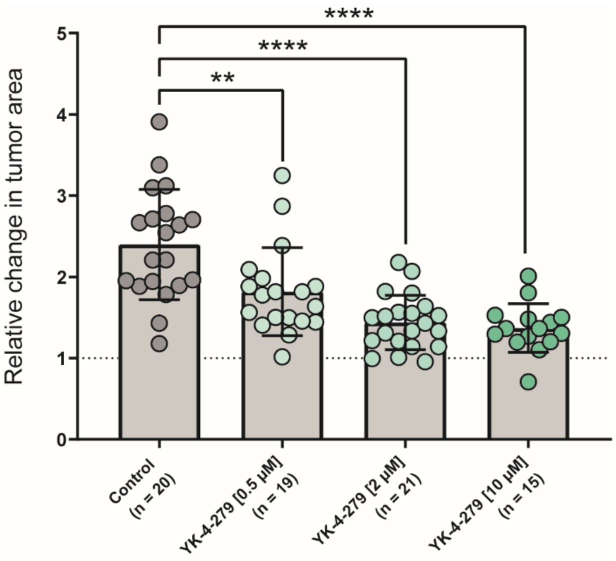

Supplementary Figure 7: YK-4-279 concentration-dependent decrease in SK-N-MC tumor growth: Treatment of larvae xenotransplanted with GFP-labeled SK-N-MC (ShSK-E17T) cells with different concentrations of YK-4-279 (0.5 µM n = 19, 2 µM n = 21, 10 µM n = 15) or DMSO (n = 20). Dot plots show relative change in tumor size (3 dpi/ 1 dpi). Statistical analyses were performed with a Kruskal-Wallis test (****: $p<0.0001$, **: $p<0.005$). Error bars represent SD.

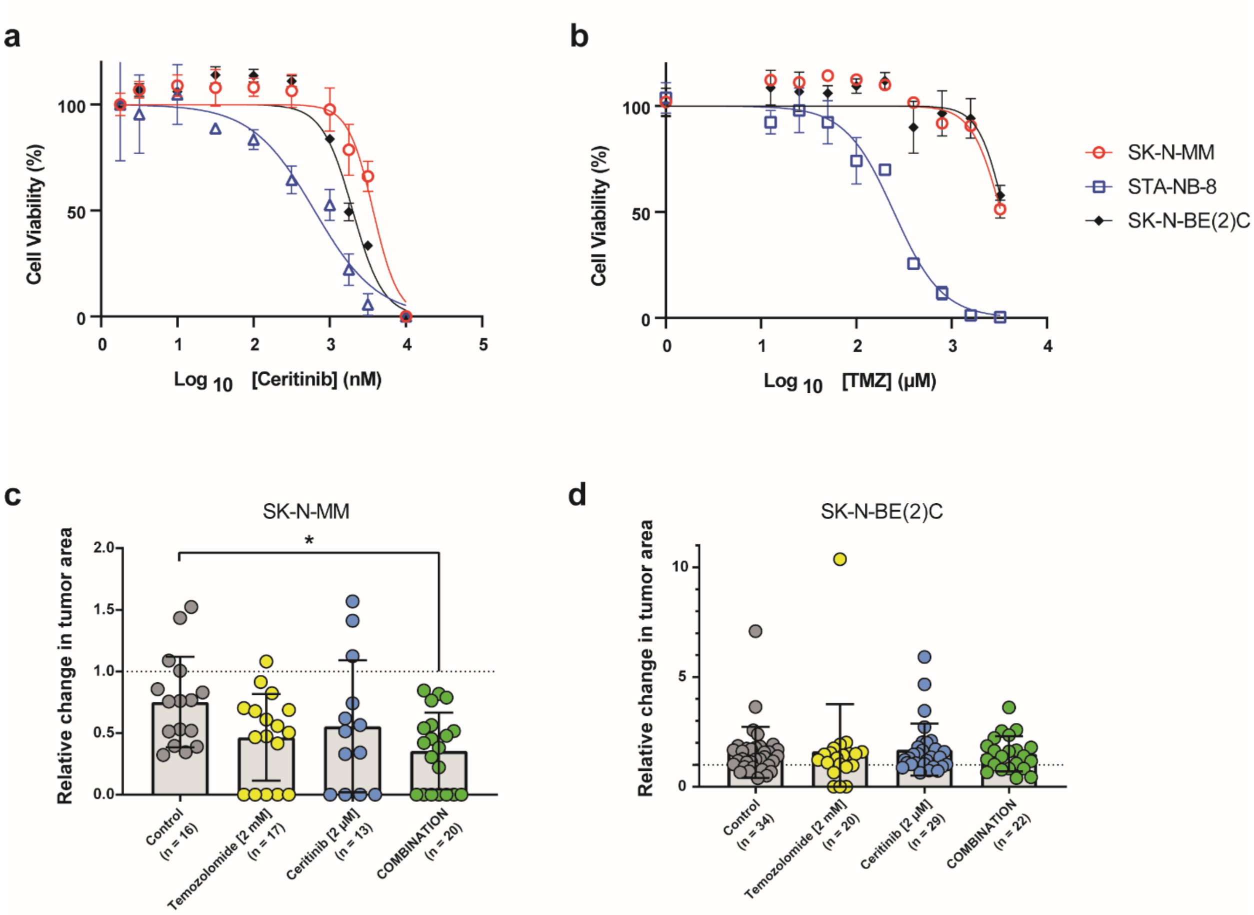

Supplementary Figure 8: Neuroblastoma compounds treatments: a-b) Neuroblastoma cells were treated with 9 concentrations of Ceritinb (a) or Temozolomide (b) for 3 days. Cell viability was determined by the CellTiterGlo assay and the respective dose-response curves were generated. Blots depict a representative example of 3 biological replicates and error bars show the SEM of the 3 technical replicates c) Treatment of larvae xenotransplanted with GFP-labeled SK-N-MM cells with 2 mM Temozolomide (n = 17), 2 µM Ceritinib (n = 13), 2 mM Temozolomide + 2 µM Ceritinib (n = 20) or DMSO (n = 16). d) Treatment of larvae xenotransplanted with GFP-labeled SK-N-BE(2)C cells with 2 mM Temozolomide (n = 20), 2 µM Ceritinib (n = 29), 2 mM Temozolomide + 2 µM Ceritinib (n = 22) or DMSO (n = 34). Dot plot show relative change in tumor size (3 dpi/ 1 dpi). Statistical analyses were performed with a Kruskal-Wallis test (*: $p<0.05$). Error bars represent SD.

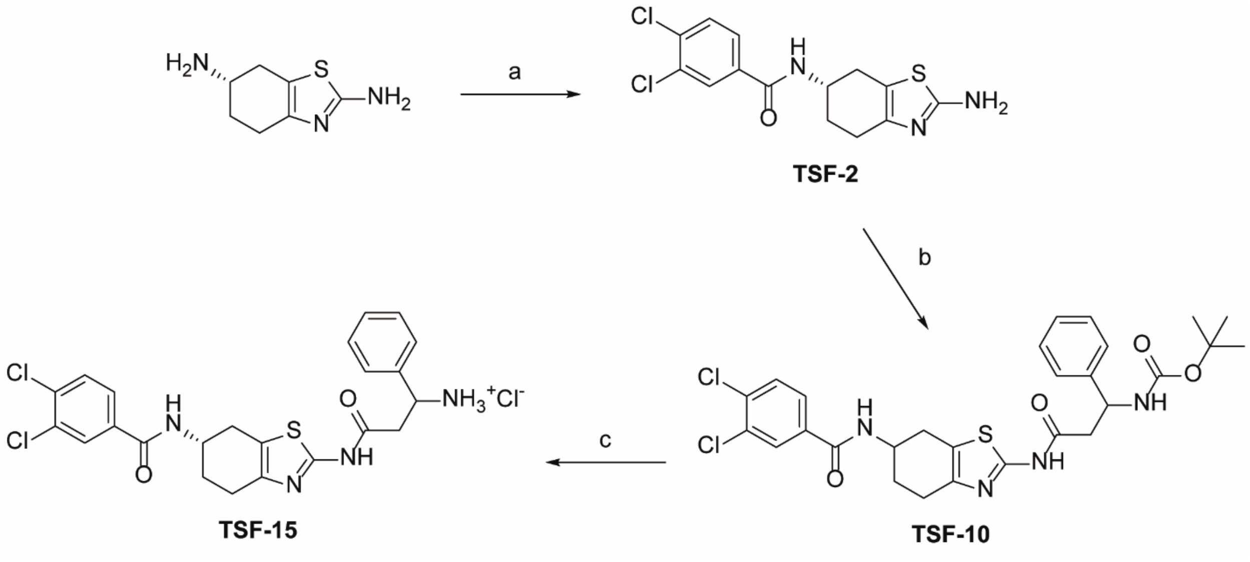

Supplementary Figure 9: Synthesis of the Hsp90 C-terminal domain inhibitor TSF-15: Compound TSF-2 was prepared by EDC/HOBt-promoted amide coupling of 3,4-dichlorobenzoic acid with the aliphatic 6-amino group of (*S*)-4,5,6,7-tetrahydrobenzo[*d*]thiazole-2,6-diamine. In the next step, 3-((*tert*-butoxycarbonyl)amino)-3-phenylpropanoic acid was coupled to the aromatic 2-amino group of TSF-2 to prepare compound TSF-10. In the last step, final compounds TSF-15 was prepared by removal of the Boc-protecting group of TSF-10 by acidolysis.

Reagents and conditions. (a) (i) 3,4-dichlorobenzoic acid, DMF, EDC, HOBt, NMM, 0 °C, 15 min, (ii) (*S*)-4,5,6,7-tetrahydrobenzo[*d*]thiazole-2,6-diamine, (b) (i) 3-((*tert*-butoxycarbonyl)amino)-3-phenylpropanoic acid, DMF, EDC, HOBt, NMM, 0 °C, 15 min, (ii) TSF-2, 20 °C, overnight; (c) 4 M HCl in 1,4-dioxane, 1,4-dioxane, 20 °C, overnight.

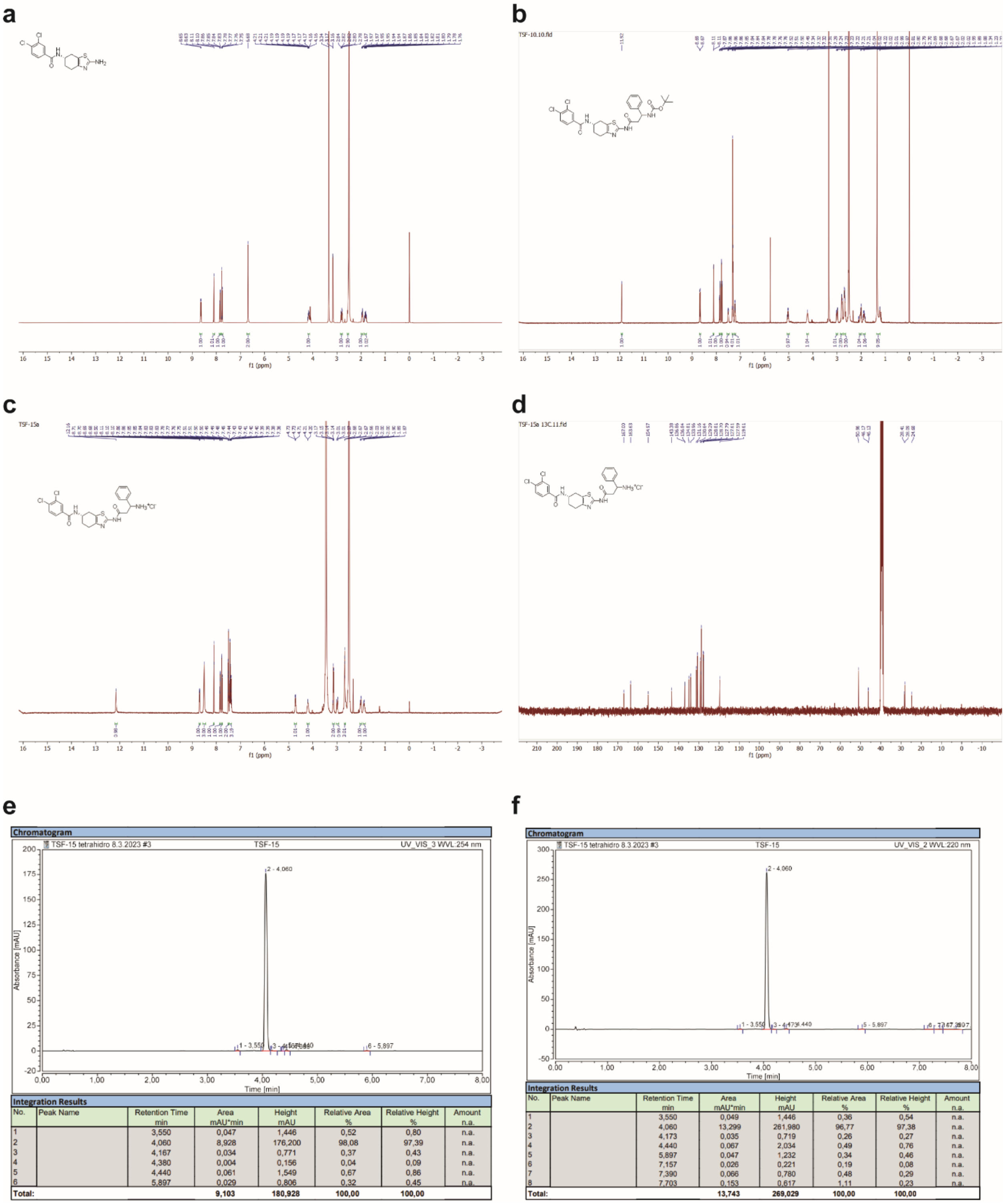

No.	Peak Name	Retention Time min	Area mAU*min	Height mAU	Relative Area %	Relative Height %	Amount n.a.
1		3,550	0,047	1,446	0,52	0,80	n.a.
2		4,060	8,928	176,200	98,08	97,39	n.a.
3		4,167	0,034	0,771	0,37	0,43	n.a.
4		4,380	0,004	0,156	0,04	0,09	n.a.
5		4,440	0,061	1,549	0,67	0,86	n.a.
6		5,897	0,029	0,806	0,32	0,45	n.a.
Total:			9,103	180,928	100,00	100,00	

No.	Peak Name	Retention Time min	Area mAU*min	Height mAU	Relative Area %	Relative Height %	Amount n.a.
1		3,550	0,049	1,446	0,36	0,54	n.a.
2		4,060	13,299	261,980	96,77	97,38	n.a.
3		4,173	0,035	0,719	0,26	0,27	n.a.
4		4,440	0,067	2,034	0,49	0,76	n.a.
5		5,897	0,047	1,232	0,34	0,46	n.a.
6		7,157	0,026	0,221	0,19	0,08	n.a.
7		7,390	0,066	0,780	0,48	0,29	n.a.
8		7,703	0,153	0,617	1,11	0,23	n.a.
Total:			13,743	269,029	100,00	100,00	

Supplementary Figure 10: Characterisation of compounds. a) ^{1}H NMR (400 MHz, DMSO-d_6) spectrum of TSF-2; b) ^{1}H NMR (400 MHz, DMSO-d_6) spectrum of TSF-10; c) ^{1}H NMR (400 MHz, DMSO-d_6) spectrum of TSF-15; d) ^{13}C NMR (101 MHz, DMSO-d_6) spectrum of TSF-15; e) UPLC chromatogram of TSF-15 at 254 nm; f) UPLC chromatogram of TSF-15 at 220 nm.

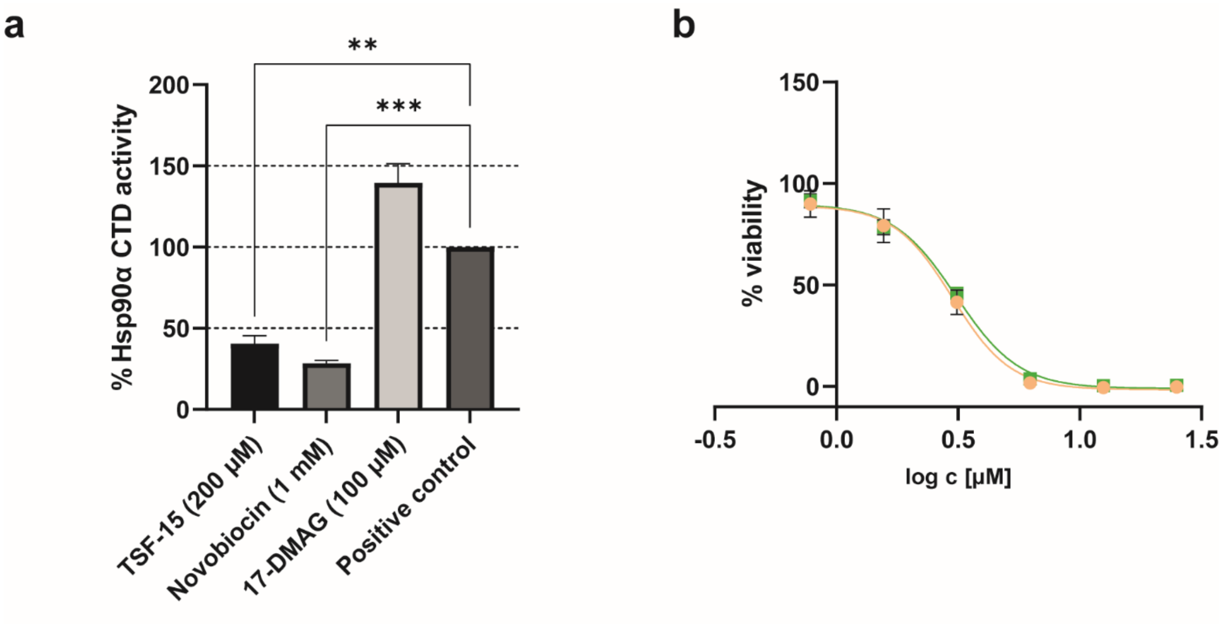

Supplementary Figure 11: Biological evaluation of Hsp90 CTD inhibitor TSF-15. a) An Hsp90α CTD inhibitor screening assay was performed to determine whether TSF-15 was able to inhibit the binding of Hsp90 CTD to its target protein cyclophilin D (PPID), as measured by TR-FRET. TSF-15 showed inhibition of Hsp90 CTD at 200 µM (**: $p<0.01$). Novobiocin, a known Hsp90 CTD inhibitor, inhibited Hsp90 CTD binding to PPID at 1 mM (***: $p<0.001$), while the Hsp90 NTD inhibitor 17-DMAG was inactive as expected. Statistical analysis was performed using one way ANOVA post hoc Dunnett's test. Error bars represent SD. b) Compound TSF-15 showed antiproliferative activity in the Ewing sarcoma SK-N-MC cell line (SKshctrl) with IC_{50} values of 3.05±0.08 µM. Error bars represent SD.

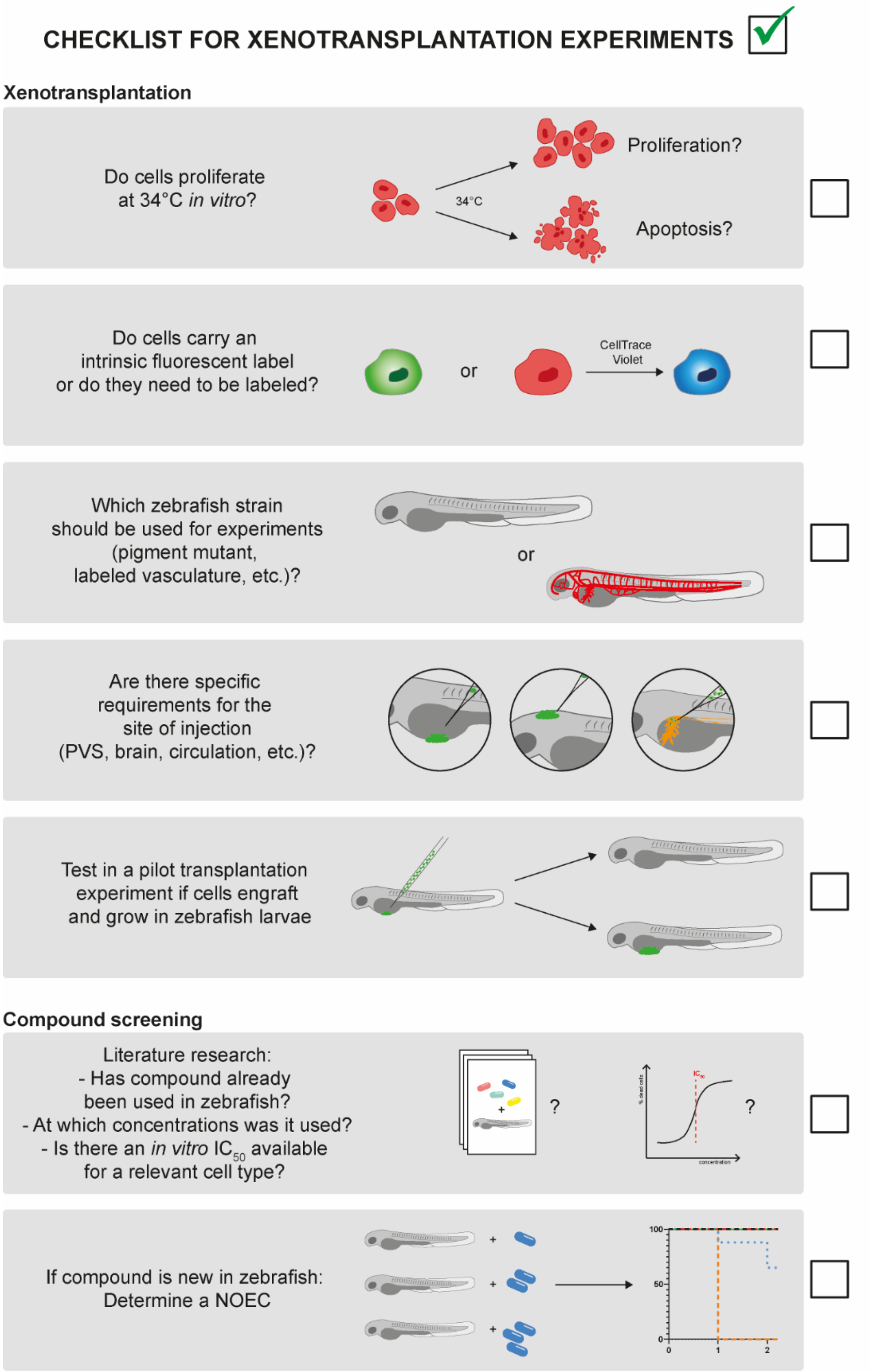

Supplementary Figure 12: Checklist for xenotransplantation experiments
